# Atropisomeric [(diphosphine)Au_2_Cl_2_] Complexes and their Catalytic Activity Towards Asymmetric Cycloisomerisation of 1,6-Enynes

**DOI:** 10.1002/chem.201404496

**Published:** 2014-12-11

**Authors:** Elena M Barreiro, Ekaterina V Boltukhina, Andrew J P White, King Kuok (Mimi) Hii

**Affiliations:** [a] Department of Chemistry, Imperial College London Exhibition Road, South Kensington, London SW7 2AZ (UK) E-mail: mimi.hii@imperial.ac.uk

**Keywords:** aurophilicity, catalysis, cycloisomerization, enynes, gold

## Abstract

X-ray crystal structures of two [(diphosphine)Au_2_Cl_2_] complexes (in which diphosphine=P-Phos and xylyl-P-Phos; P-Phos=[2,2′,6,6′-Tetramethoxy-4,4′-bis(diphenylphosphino)-3,3′-bipyridine]) were determined and compared to the reported structures of similar atropisomeric gold complexes. Correlations between the Au⋅⋅⋅Au distances and torsional angles for the biaryl series of ligands (MeOBIPHEP, SEGPhos, and P-Phos; BIPHEP=2,2′-bis(diphenylphosphino)-1,1′-biphenyl, SEGPhos=[(4,4′-bi-1,3-benzodioxole)-5,5′-diyl]bis[diphenylphosphine]) can be made; these measurements appear to be very dependent upon the phosphorous substituent. Conversely, the same effect was not observed for ligands based on the binaphthyl (BINAP) series. The catalytic activity of these complexes was subsequently assessed in the enantioselective cycloisomerisation of 1,6-enynes and revealed an over-riding electronic effect: more-electron-rich phosphines promote greater enantioselectivity. The possibility of silver acting as a (co-)catalyst was ruled out in these reactions.

## Introduction

The recent upsurge of interest to explore the medicinal properties of three-dimensional molecular structures and fragments[1–4] has driven the need for synthetic methodologies that can generate complex scaffolds from acyclic precursors in a single step. An exemplar of such reactions is the cycloisomerisation of 1,6-enynes, which generates carbo- and heterobicyclo[4.1.0]heptene derivatives with up to three stereocentres, thus provides extremely valuable compounds for organic synthesis.[5–7]

**Scheme 1:**
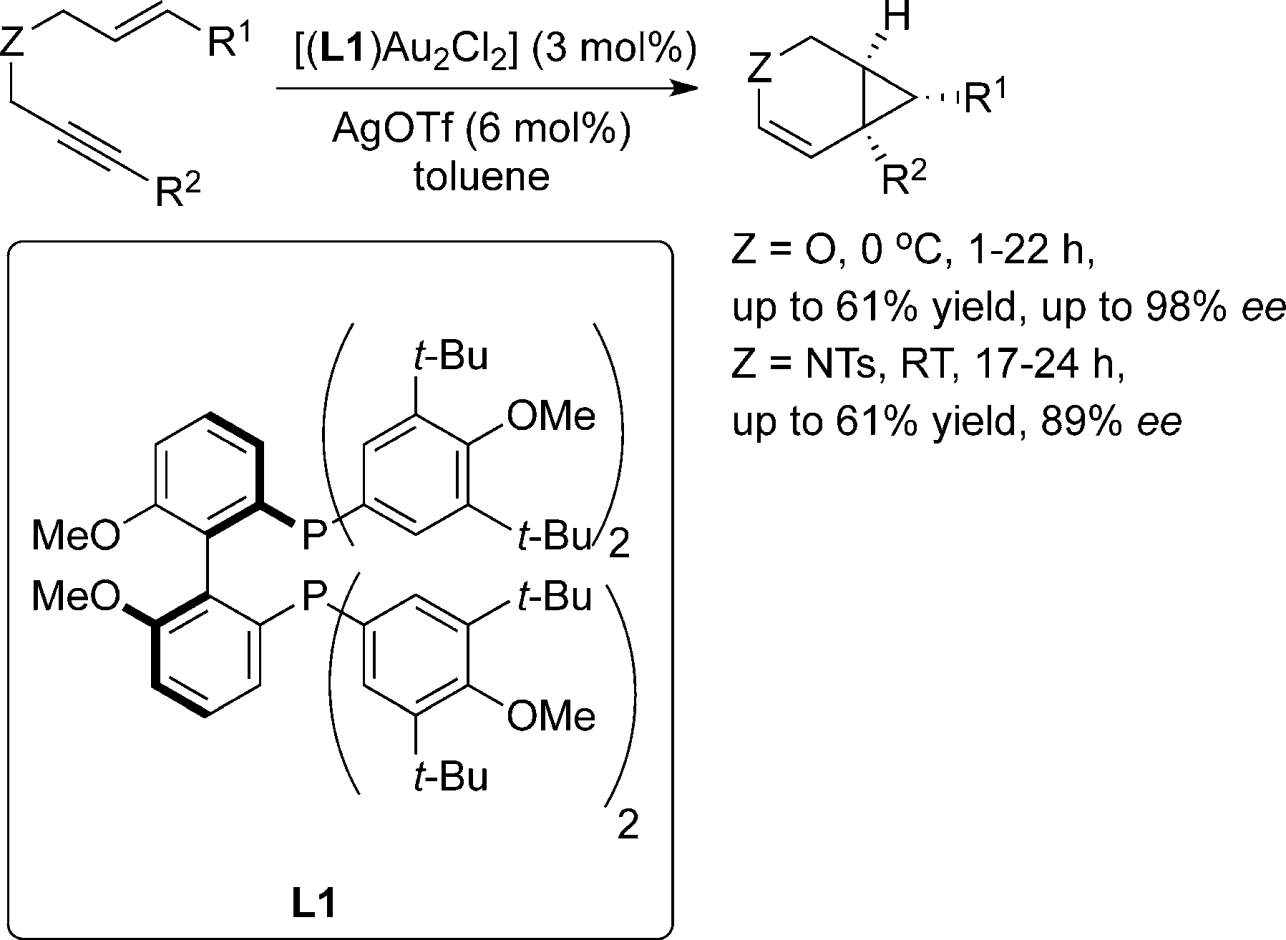
Enantioselective Au-catalysed cycloisomerisation of 1,6-enynes [Z=O, *N*-Tosyl (Ts)].

The reaction was first discovered in 1995 by Blum and co-workers by using a catalytic amount of PtCl_4_.[8] Since then, a number of other π-acidic transition-metal catalysts were reported, including Rh,[9, 10] Ir,[11] Pt[12–14] and Au catalysts.[15, 16] The first enantioselective examples were provided by Michelet and co-workers in 2009, by using a gold(I) complex of the chiral diphosphine ligand **L1** (Scheme 1).[17, 18] Subsequently, the enantiomeric excess (*ee*) values obtained have been superseded by other transition-metal catalysts (Pt,[19] Rh[20–22] and Ir[23]), but gold is the only reported system that is catalytically active at room temperature.

**Scheme 2:**
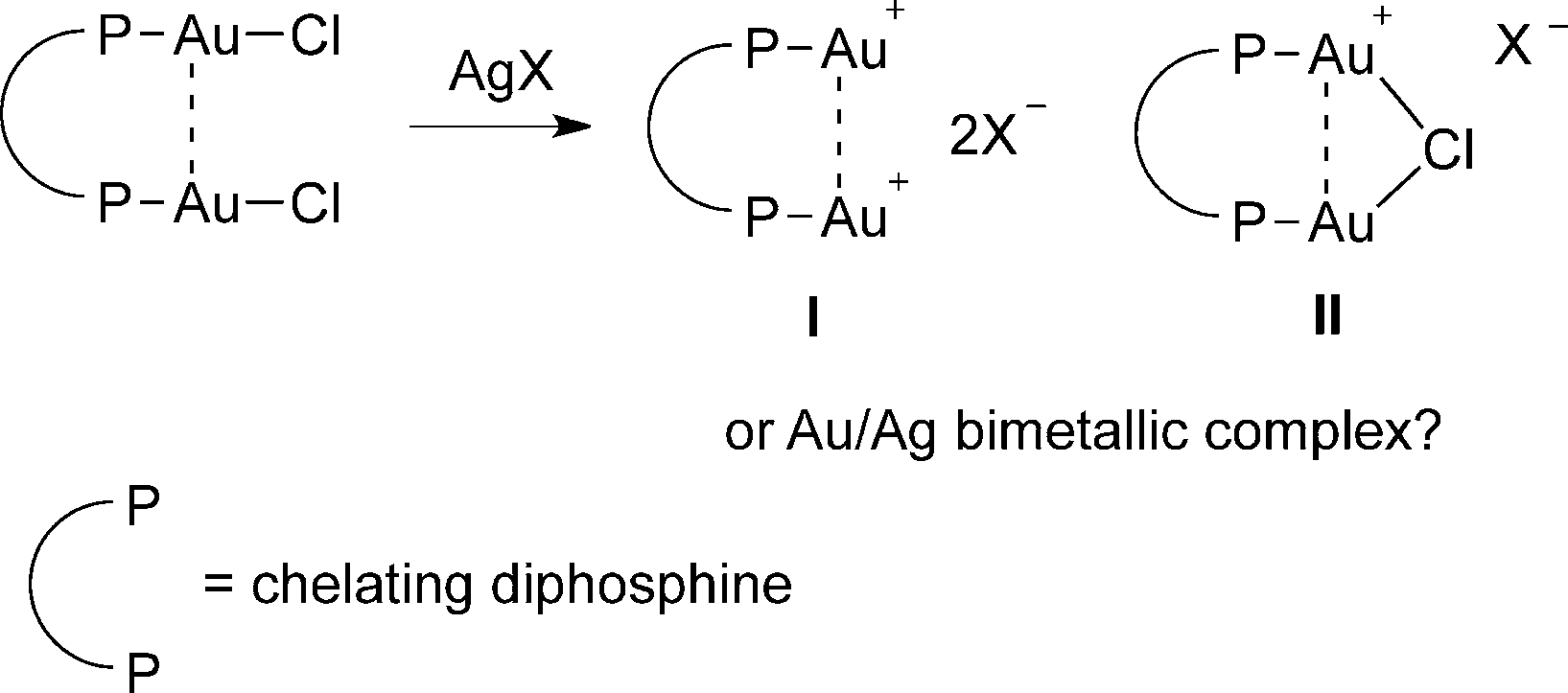
Formation of cationic gold complexes.

The catalytic activity of cationic gold complexes continues to be a subject of considerable intrigue and debate.[24] Diphosphine ligands were frequently found to be effective for many asymmetric transformations catalysed by gold. More often than not, silver salts are required to activate [(diphosphine)Au_2_Cl_2_] precursors towards catalysis. This is thought to generate a cationic species **I** (Scheme 2) with sufficient π-Lewis acidity for catalysis, but the characterisation of such cationic gold complexes remains elusive; no X-ray crystal structure has been determined yet. Most diphosphine ligands do not form chelate rings with gold(I), instead linear geometries that may contain short gold–gold distances, indicative of aurophilic interactions, are favoured.[25] Thus, a significant gold–gold cooperative effect may be important for the catalytic activity. In reactions catalysed by [AuCl(PR_3_)]/AgX systems, chloro-bridged species **II** (Scheme 2) has been isolated and identified. Also, the participation of silver in the catalytic reaction (presumably via bimetallic complexes) cannot be ruled out.[26, 27]

In this paper, we report the results of a preliminary study conducted with different diphosphine–gold(I) complexes for the cycloisomerisation reactions of 1,6-enynes. We have attempted to correlate the structural parameters of the gold complexes to the observed enantioselectivity and to investigate the role of the silver salt on the outcome of these reactions.

## Results and Discussion

The study began with a search in the CCDC database for reported structures of chiral atropisomeric biaryl [(diphosphine)Au_2_Cl_2_] complexes, from which 14 discreet structures corresponding to 9 compounds can be found (diphosphine=**L1**–**L9**, Table 1).[28] The major conformational parameters that vary between the complexes are given in Table 1; namely, the torsional twist about the central aryl–aryl bond (*θ*), the Au–Cl bond length and the intramolecular Au⋅⋅⋅Au separation.

**Table 1:** Selected structural parameters for [(diphosphine)Au_2_Cl_2_] complexes

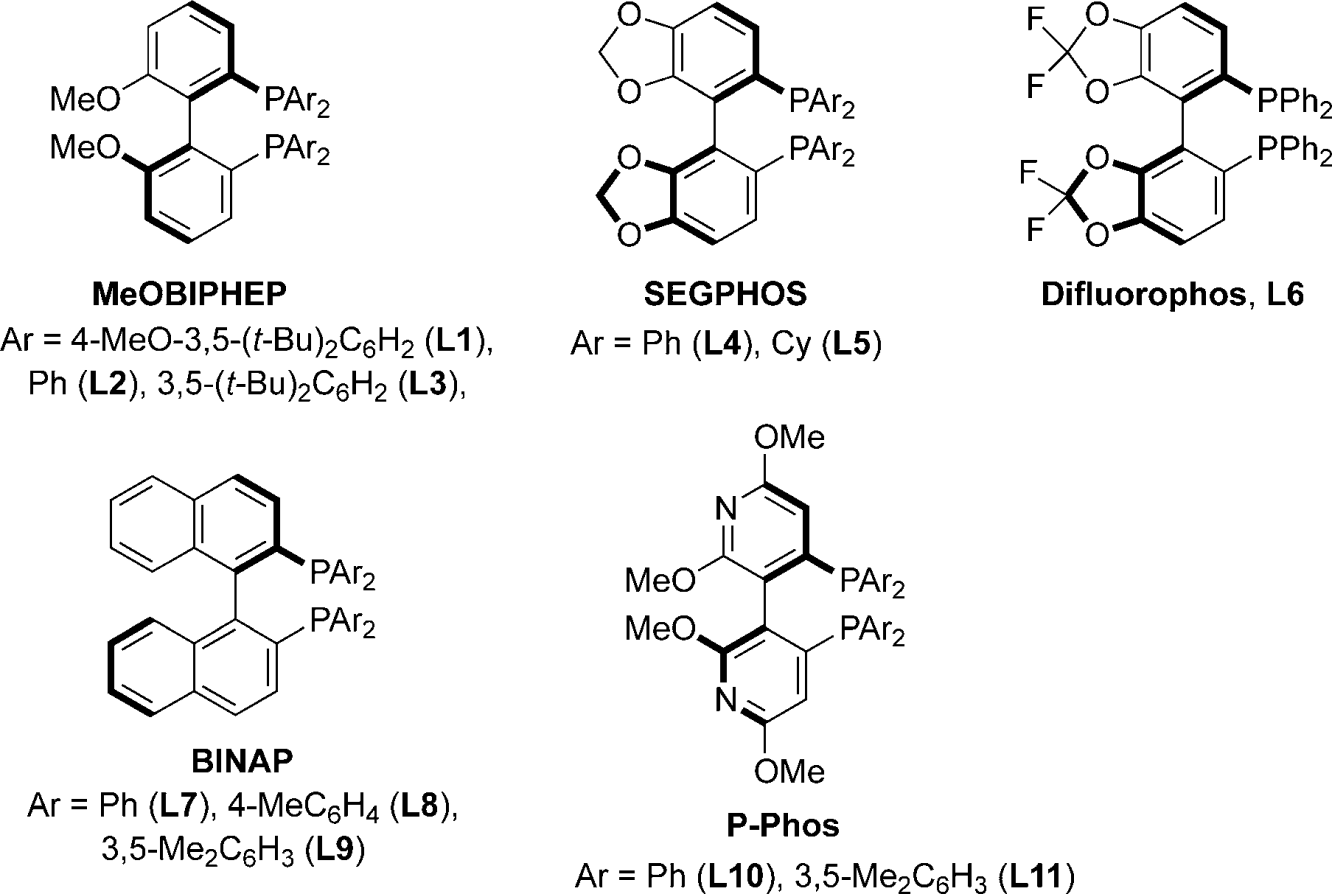					
Diphosphine	Au⋅⋅⋅Au [Å]	Au–Cl [Å]	*θ*^[a]^ [°]	CCDC code	Reference
**L1**	5.323	2.266, 2.269	104.27	AJOHIP	[29]
	5.316	2.275	105.74	AJOHIR01	[30]
	5.296	2.290, 2.283	103.62	AJOHIP02	[31]
**L2**	5.820	2.282	105.05	EKOLIA	[32]
	3.147	2.2932	95.58	ELOCUE	[31]
**L3**	4.131	2.263	96.54	YUZLUB	[33]
	4.984	2.275, 2.274	99.58		
**L4**	2.994	2.292, 2.279	68.08	TADSIC	[30]
**L5**	5.661	2.286, 2.289	114.47	LITKAB	[34]
**L6**	3.785	2.278	101.42	ELOCOY	[31]
**L7**	5.482	2.290	98.50	KIKBAI	[35]
	5.422	2.282	98.44	NUXBUE	[36]
	5.480	2.277, 2.299	90.68	NUXCAL	[36]
**L8**	5.976	2.288	110.58	YAQGIH	[37]
**L9**	5.466	2.303, 2.284	94.98	NANPUP	[38]
**L10**	3.670	2.277, 2.282	88.93	–	[b]
	3.182	2.290, 2.295	102.76		
**L11**	4.792	2.281, 2.274	114.20	–	[b]

[a] Dihedral angle of the biaryl backbone, C2-C1-C1′-C2′ torsion angle, obtained from crystal structures of the [(diphosphine)Au_2_Cl_2_] structures. [b] This work.[39]

**Figure 1:**
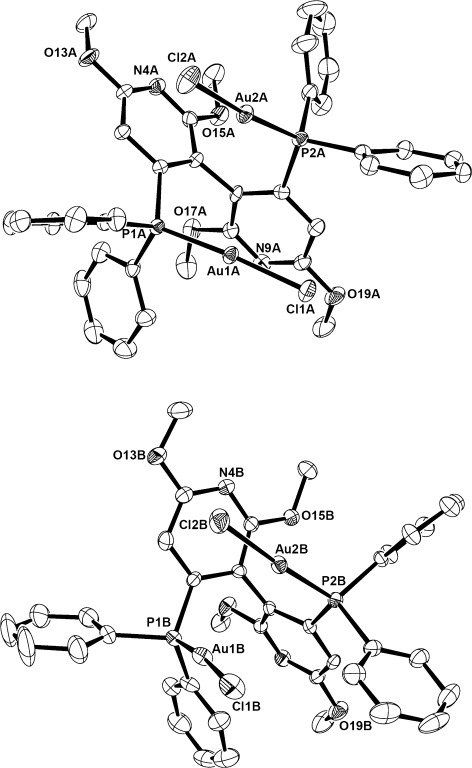
Structures of the two independent molecules present in the crystals of [(L10)Au_2_Cl_2_], A (top) and B (bottom). Hydrogen atoms are omitted for clarity.

**Figure 2:**
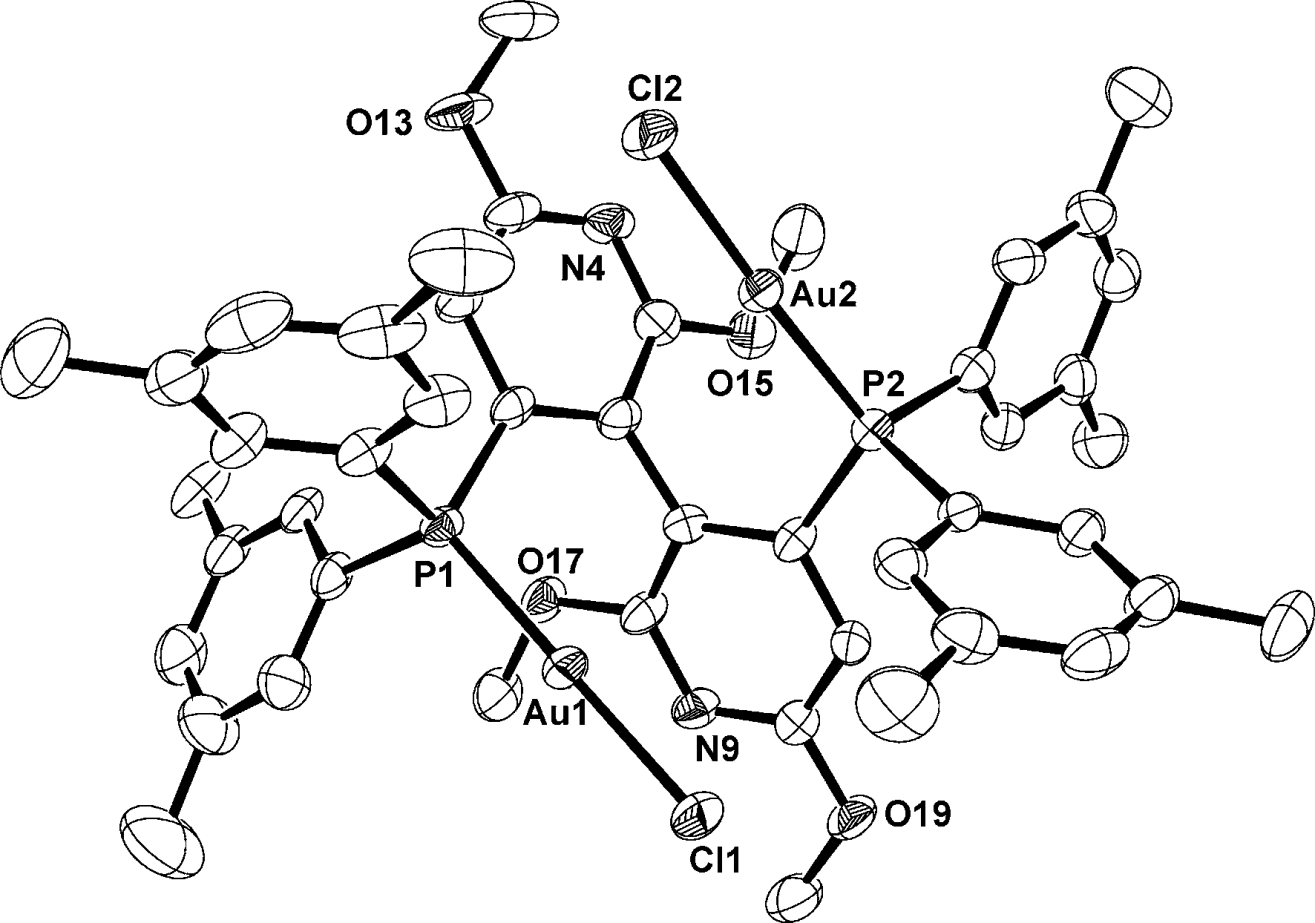
Crystal structure of [(L11)Au_2_Cl_2_]. Hydrogen atoms and co-crystallisation solvents are omitted for clarity.

Additionally, two complexes that contained P-Phos (**L10**) and xylyl-P-Phos (**L11**) were also prepared and their structures were determined by X-ray crystallography (Figures 1 and 2). Complex [(**L10**)Au_2_Cl_2_] was found to crystallise with two independent molecules (A and B) in the asymmetric unit (Figure 1). The compositions of [(**L10**)Au_2_Cl_2_] (Figure 1) and [(**L11**)Au_2_Cl_2_] (Figure 2) differ only in the substituents on the phosphorus atoms; phenyl in [(**L10**)Au_2_Cl_2_] and 3,5-dimethylphenyl in [(**L11**)Au_2_Cl_2_], yet all three molecules have noticeably different conformations.

The two independent molecules of [(**L10**)Au_2_Cl_2_] have similar twists about the central π–π bond (≍88° and 82°), whereas that for [(**L11**)Au_2_Cl_2_] is substantially different (≍66°). For [(**L10**)Au_2_Cl_2_]-A the orientation of the P–Au bond relative to its adjacent pyridyl ring is the same in both instances (≍47°), whereas in [(**L10**)Au_2_Cl_2_]-B they differ markedly (≍88° and 29° for P1 and P2, respectively). In [(**L11**)Au_2_Cl_2_] the twists are again similar to each other, but at approximately 25° they are very different to those seen in [(**L10**)Au_2_Cl_2_]-A. As a consequence, both [(**L10**)Au_2_Cl_2_]-A and [(**L11**)Au_2_Cl_2_] have approximate *C*_2_ symmetry, whereas [(**L10**)Au_2_Cl_2_]-B does not. The consequence of these differing twists is markedly varied intramolecular Au1⋅⋅⋅Au2 separations of 3.6701(3), 3.1821(2) and 4.7921(4) Å in [(**L10**)Au_2_Cl_2_]-A, [(**L10**)Au_2_Cl_2_]-B and [(**L11**)Au_2_Cl_2_], respectively (Table 1).

**Figure 3:**
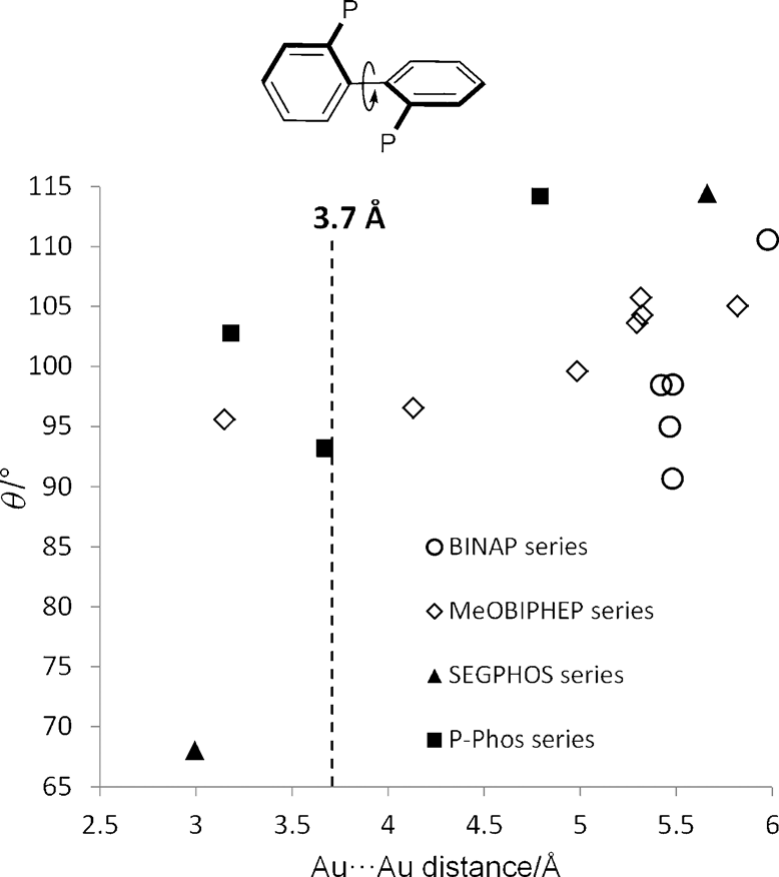
Scatter plot of torsional angles between axially-chiral aromatic rings (*θ*) [°] against the Au⋅⋅⋅Au distances [Å] for atropisomeric diphosphine–Au complexes. BINAP series (○), MeOBIPHEP series (◊), SEGPHOS series (▴), P-Phos series (▪).

Manifestation of aurophilicity is generally indicated by Au⋅⋅⋅Au distances that are shorter than the sum of two van der Waals radii of 3.7 Å.[40] For the atropisomeric diphosphine ligands (MeOBIPHEP (**L1**–**L3**), BINAP (**L7**–**L9**), SEGPHOS (**L4**–**L5**) and P-Phos) it is expected that the Au⋅⋅⋅Au distance is dependent upon the degree of rotational freedom between the aromatic rings that defines the axial chirality, which is, in turn, determined by the nature of the biaryl that constitutes the scaffold, as well as the P substituents. With this in mind, a scatter plot of the torsional angle between the two central aryl rings against the Au⋅⋅⋅Au distance was produced (Figure 3) and revealed some interesting trends.

Greater conformational flexibility was found in the biaryl series MeOBIPHEP, SEGPHOS and P-Phos, for which it is common to find more than one independent molecule for each complex. Nevertheless, the Au⋅⋅⋅Au distance tends to increase with a greater torsional angle between the two aromatic rings. Within the MeOBIPHEP series (**L1**–**L3**, ◊, Figure 3) the introduction of *tert*-butyl and methoxy substituents on the P-aryl group appear to augment both of these values. The difference between **L1** and **L3** is particularly notable: the presence of an additional methoxy substituent at the *para* position appears to lengthen the Au⋅⋅⋅Au distance quite significantly. The same trend can be observed in the P-Phos series (**L12** and **L13**, ▪, Figure 3), in which the presence of electron-donating xylyl groups increased the Au⋅⋅⋅Au distance. For the SEGPHOS series (**L4** and **L5**, ▴, Figure 3), substitution of PPh_2_ by PCy_2_ (Cy=cyclohexyl) groups also has a dramatic effect on the intermetallic distance. As a result, Au⋅⋅⋅Au distances of <3.7 Å were only observed in four of these biaryl diphosphine ligated complexes, which all contained PPh_2_ substituents. Thus, it appeared that intermetallic interactions are discouraged by more-electron-rich P donors.

In contrast, Au⋅⋅⋅Au distances in the gold complexes of the BINAP series (**L7**–**L9**) are essentially insensitive to the P substituent (○, Figure 3). This is attributed to the higher barrier of rotation for the binaphthyl ring. For this particular series of gold complexes, all Au⋅⋅⋅Au distances were found to be >5.4 Å. These observations suggest that aurophilicity is relatively uncommon for these atropisomeric [(diphosphine)Au_2_Cl_2_] complexes, inhibited by more-electron-rich P-donor groups and by restriction of the conformational flexibility of the aryl–aryl bond.

**Scheme 3:**
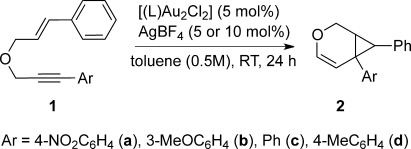
Au-catalysed cycloisomerisation of 1,6-enynes 1 a–d.

In the next phase of this study, the catalytic activity of selected complexes was tested in the cycloisomerisation of ether substrates **1 a**–**d** (Scheme 3). Selected results are presented in Table 2.

**Table 2:** Effect of chiral diphosphine ligands and Au/Ag stoichiometry in the cycloisomerisation of 1,6-enynes (Scheme 3)^[a]^

Entry	Substrate	L	Au/Ag	Yield^[b]^ [%]	*ee*^[c]^ [%]
1	**1 a**	**(*S*)-L1**	1:1	87	99 (+)
2	**1 a**	**(*R*)-L7**	1:1	70	46 (−)
3	**1 a**	**(*R*)-L8**	1:1	85	75 (−)
4	**1 a**	**(*R*)-L10**	1:1	96	38 (−)
5	**1 a**	**(*R*)-L11**	1:1	92	60 (−)
6	**1 a**	**(*R*)-L11**	2:1	89	64 (−)
7	**1 a**	–	0:1	–	–
8	**1 b**	**(*R*)-L11**	1:1	86	66 (−)
9	**1 b**	**(*R*)-L11**	2:1	54	64 (−)
10	**1 c**	**(*R*)-L11**	1:1	76	78 (+)
11	**1 c**	**(*R*)-L11**	2:1	69	74 (+)
12	**1 d**	**(*R*)-L11**	1:1	54	72 (+)
13	**1 d**	**(*R*)-L11**	2:1	45	74 (+)

[a] Reaction conditions: Substrate **1** in toluene (0.5 m), [Au]_2_ (5 mol %), [Ag] (5 or 10 mol %), RT, 24 h. [b] Isolated yield. [c] Determined by chiral HPLC. Optical activity of the product is given in parentheses.

The cyclisation of ether-tethered enyne substrate **1 a** was examined initially as the model reaction, performed in toluene at room temperature. The product was somewhat unstable, therefore quick purification by flash chromatography was necessary to achieve good isolated yields. The benchmark for this study was set by reproduction of the result reported by Michelet and co-workers by using ligand **L1**:[17, 18] the product **2 a** can be obtained in 87 % yield with 99 % *ee* (Table 2, entry 1). Guided by the structural analysis (Table 1), two complexes from the BINAP series (**L7** and **L8**) were tested as catalysts because they have similar Au⋅⋅⋅Au distances (>5.3 Å). In both cases, the product was isolated in good yields. By using the BINAP ligand **L7**, a modest 46 % *ee* was obtained, whereas tolyl-substituted **L8** gave a much higher 75 % *ee* (Table 1, entries 2 and 3). Similarly, two ligands from the P-Phos series (**L10** and **L11**) were selected for their similar torsional angles to **L1**. In these cases, excellent yields of the product were obtained. Once again, the more-electron-rich *P*-xylyl-substituted complex **L11** offered significantly better enantioselectivity than **L10** (38 vs. 60 % *ee*; Table 1, entries 4 and 5).

Correlations between structural parameters obtained from solid-state structures and homogeneous catalytic reactions should always be exercised with caution. Nevertheless, these preliminary studies seem to suggest that the enantioselectivity of atropisomeric biaryl diphosphine ligands can be enhanced by using more-electron-rich P substituents.

**Scheme 4:**
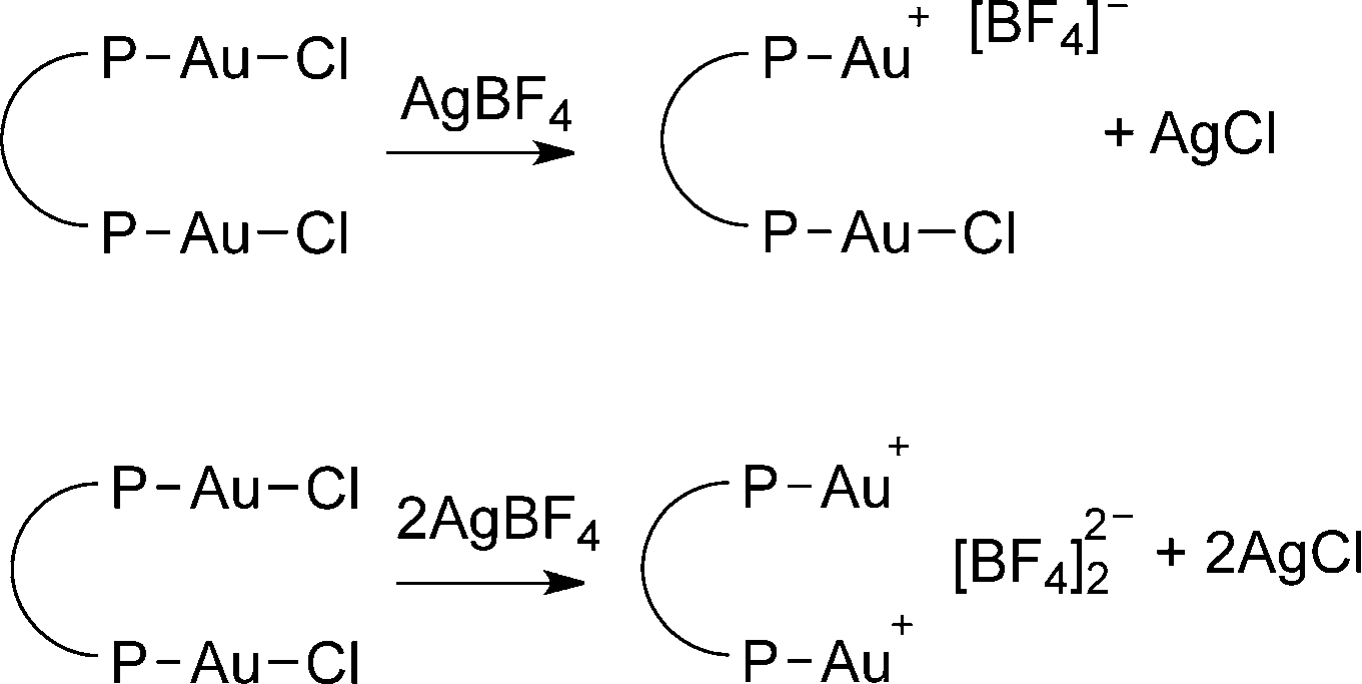
Possible complexes generated by using different Au/Ag ratios.

Next, we examined the importance of the silver salt in these reactions. In previous studies, the choice of silver salt/counter-anion often had a profound effect on the catalytic activity and it has been suggested that the participation of silver catalysis cannot be discounted in such reactions.[26] In this case, a control reaction showed that the linear substrate **1 a** remained unchanged in the presence of AgBF_4_ alone at ambient temperature (Table 2, entry 7), that is, any background racemic process due to the silver salt can be categorically ruled out. Subsequently, by using the **L11**-ligated complex, the cycloisomerisations of three other substrates **1 b**–**d** were performed in the presence of various amounts of silver salt, with the stoichiometric ratio of Au/Ag either 1:1 or 2:1 (Table 2, entries 8–12). In each case, the product yield was lower when silver salt (0.5 equiv) was employed but the level of enantioselectivity remained invariant. This is a significant observation. Considering the different cationic complexes that can be generated in each case (Scheme 4), the involvement of a bimetallic Au/Ag complex as the active catalyst can be ruled out because this can only be generated in a significant quantity when a sub-stoichiometric amount of silver salt is used.[41] Furthermore, the result also strongly suggests that each metal centre operates independently as an active catalytic site, that is, the presence of any cooperative effect between the two proximal Au metal centres may be discounted. This is commensurate with the previous study, in which greater enantioselectivity was observed with complexes that had larger intermetallic distance/less propensity to form Au⋅⋅⋅Au interactions for steric (**L1**) and/or electronic (**L8**, **L11**) reasons.

Finally, the active catalyst was also generated in situ by pre-mixing the gold–chloride complex with the silver additive prior to addition of the substrate, or by introducing an intermediate operation, in which the AgCl formed was removed by filtration through a bed of Celite before the introduction of the substrate. These procedures had no effect on the results. Hence, the involvement of [L–Au–Cl–Ag]^+^ is also highly unlikely.

## Conclusion

We have conducted a structural study on a series of [(diphosphine)Au_2_Cl_2_] complexes. Even within the small selection of examples, some trends can be observed within the diaryl diphosphine complexes, for instance an increase in the electron-donating character of the P-Ar substituent increases the Au⋅⋅⋅Au distance. This effect is not observed, however, in the BINAP series of ligands, which is attributed to the greater barrier to rotation/more-rigid structure. The catalytic activity of selected complexes was tested in the cycloisomerisation of 1,6-enynes; the incorporation of electron-rich P donors enhanced the stereoselectivity. Critically, the enantioselectivity of the process was not affected by using different gold/silver ratios or by removal of the silver by-product. This suggests that each metal centre is likely to operate independently and that silver does not participate in the process. These observations are useful for the future design of better catalysts for these reactions.

## Supporting Information

As a service to our authors and readers, this journal provides supporting information supplied by the authors. Such materials are peer reviewed and may be re-organized for online delivery, but are not copy-edited or typeset. Technical support issues arising from supporting information (other than missing files) should be addressed to the authors.

miscellaneous_information
