# Supplementary material for: Atropisomeric [(diphosphine)Au2Cl2] Complexes and their Catalytic Activity Towards Asymmetric Cycloisomerisation of 1,6-Enynes
Source: Chemistry. 2014 Dec 11;21(6):2686–90. doi: 10.1002/chem.201404496 (PMC4524423; doi:10.1002/chem.201404496)
Supplement: Supplementary file 1 — miscellaneous_information [file chem0021-2686-sd1.pdf]

CHEMISTRY
A European Journal

Supporting Information

© Copyright Wiley-VCH Verlag GmbH & Co. KGaA, 69451 Weinheim, 2015

Atropisomeric [(diphosphine)Au_2Cl_2] Complexes and their Catalytic Activity Towards Asymmetric Cycloisomerisation of 1,6-Enynes

Elena M. Barreiro,[a] Ekaterina V. Boltukhina,[b] Andrew J. P. White,[a] and King Kuok (Mimi) Hii*[a]

chem_201404496_sm_miscellaneous_information.pdf

Supporting Information

Experimental Section p.1
- General p.1
- Synthesis of gold(I)-chloride complexes p.1
- Synthesis of the substrates p.2
- Platinum-catalysed racemic cycloisomerisation p.5
- Gold(I)-catalysed enantioselective cycloisomerisation p.5

Crystal Structures p.8

NMR Spectra p.12

Experimental Section

General

Solvents were dried by passing through the columns of molecular sieves in a solvent purification system. Unless otherwise stated, all reagents and catalysts were procured commercially and used as received. Catalytic reactions were performed under a dry nitrogen atmosphere in glassware covered with aluminium foil. Flash column chromatography and TLC were performed on silica gel (Kieselgel 60). TLC were visualised with molybdate dip or UV light. NMR spectra were obtained on Bruker AVACE400 spectrometer with ^1H at 400 MHz, ^{31}P at 161.9 MHz, and ^{13}C at 100.6 MHz. Chemical shifts are reported in δ (ppm), referenced to the residual protons and ^{13}C signals of deuterated chloroform for ^1H and ^{13}C NMR spectra, respectively. Multiplicity is abbreviated to s (singlet), d (doublet), t (triplet), q (quartet) and m (multiplet). HPLC analyses were performed on Hewlett Packard HPLC machines (HP1050). Melting points were determined using an Electrothermal Gallenhamp apparatus fitted with a calibrated thermometer with an error of ±2°C. Infrared spectra were recorded using a Perkin Elmer Spectrum 100 FT-IR spectrometer equipped with a beam-condensing accessory. $[\alpha]_D$ values were determined on an Optical Activity Ltd polarimeter using a 1cm cell with c (concentration) in mg/mL. High-resolution mass spectra (HRMS) were recorded on Micromass Autospec Premier, Micromass LCT Premier, or VG Platform II spectometer using CI or ESI. Elemental analyses were performed by the Analytical Services at London Metropolitan University (UK).

Synthesis of gold(I)-chloride complexes

General procedure: Thiodiglycol (3 mol equiv.) was added dropwise to an ice-cold, stirred solution of $NaAuCl_4{\cdot}H_2O$ (1 mol equiv.) in water. When the orange solution turned transparent, a solution of the corresponding phosphine **L** (0.5 mol equiv.) in $CHCl_3$ was added and stirring continued. After 3 h, the organic layer was separated, dried over $MgSO_4$ and filtered. Ethanol was added and the mixture was put into the freezer (–20°C), whereupon the requisite gold complex crystallises. Characterisation data obtained for complexes [(**L1**)Au_2Cl_2],[1] [(**L7**)Au_2Cl_2][2] and [(**L8**)Au_2Cl_2][3] were consistent with those previously reported.

[(**L10**)Au_2Cl_2] was obtained from (*R*)-P-Phos [(*R*)-(+)-2,2',6,6'-tetramethoxy-4,4'-bis(diphenylphosphino)-3,3'-bipyridine] as colourless crystals. Yield 93%; mp 255–257ºC (dec.); $[\alpha]_D^{20}$ = +207.8 (c 0.51, $CHCl_3$); ^1H NMR (400 MHz, $CDCl_3$): δ 7.65–7.34 (m, 20H, Ph), 6.30 (d, J = 11.3 Hz, 2H, CH), 3.91 (s, 6H, OMe), 3.12 (s, 6H, OMe); ^{13}C NMR (100 MHz, $CDCl_3$): δ 163.4 (d, J = 14.5 Hz), 161.7 (d, J = 14.1 Hz), 142.6, 142.0, 134.6 (d, J = 13.3 Hz), 131.7, 129.1 (d, J = 12.2 Hz), 128.9 (d, J = 12.4 Hz), 128.2 (d, J = 9.1 Hz), 127.5 (d, J = 8.7 Hz), 108.1, 54.1, 53.0; ^{31}P NMR (162 MHz, $CDCl_3$): δ +23.3; IR (neat) cm^{-1}: 2945 w, 2858 w, 1575 s, 1541 s, 1472 m, 1435 m, 1408 m, 1351 s, 1314 s, 1254 m, 1199 m, 1154 m, 1095 m, 1071 m, 1025 s, 836 m, 745 s, 711 s, 670 s, 621 m; MS (FAB) m/z (%): 1108 ($[M]^+$, 12), 1073 ($[M-Cl]^+$, 100), 841 ($[M-AuCl_2]^+$, 16), 459 (50), 307 (28); Anal. calcd. for $C_{38}H_{34}N_2O_4P_2Au_2Cl_2$: C, 41.14; H, 3.09; N, 2.52%. Found: C, 41.12; H, 3.12; N, 2.57%.

[(**L11**)Au_2Cl_2] was obtained from (*R*)-Xylyl-P-Phos [(*R*)-(+)-2,2',6,6'-tetramethoxy-4,4'-bis(di(3,5-xylyl)phosphino)-3,3'-bipyridine] as colourless crystals. Yield 95%; mp 280–281ºC (dec.); $[\alpha]_D^{20}$ = +94.1 (c 0.51, $CHCl_3$); ^{1}H NMR (400 MHz, $CDCl_3$): δ 7.11–6.92 (m, 12H, Ph), 6.27 (d, J = 11.2 Hz, 2H, CH), 3.97 (s, 6H, OMe), 3.46 (s, 6H, OMe), 2.29 (s, 12H, $PhCH_3$), 2.26 (s, 12H, $PhCH_3$); ^{13}C NMR (100 MHz, $CDCl_3$): δ 163.3 (d, J = 13.4 Hz), 161.4 (d, J = 14.3 Hz), 142.7, 142.1, 138.6 (d, J = 3.1 Hz), 138.4 (d, J = 2.8 Hz), 133.3 (d, J = 6.6 Hz), 131.8 (d, J = 14.0 Hz), 131.5 (d, J = 14.0 Hz), 129.2, 128.6, 126.8, 126.2, 108.3 (d, J = 5.0 Hz), 54.1, 53.5, 21.3 (d, J = 4.4 Hz); ^{31}P NMR (162 MHz, $CDCl_3$): δ +22.2; IR (neat) cm^{-1}: 2942 w, 2857 w, 1578 s, 1539 m, 1471 m, 1443 m, 1408 m, 1366 m, 1346 s, 1316 s, 1243 m, 1196 s, 1157 m, 1128 m, 1036 s, 992 m, 961 w, 845 s, 833 s, 686 s, 666 m; MS (FAB) m/z (%): 1220 ($[M]^+$, 10), 1185 ($[M-Cl]^+$, 100), 953 ($[M-AuCl_2]^+$, 113), 789 (42), 515 (74); Anal. calcd. for $C_{46}H_{50}N_2O_4P_2Au_2Cl_2$: C, 45.22; H, 4.13; N, 2.29%. Found: C, 45.12; H, 4.10; N, 2.31%.

Crystals suitable for X-ray crystallography were obtained by slow cystallisation from a mixture of chloroform-ethanol. Crystallographic data have been deposited at the Cambridge Crystallographic Database (CCDC), under reference codes CCDC1005640 and CCDC1005641 for (**L10**)Au_2Cl_2 and (**L11**)Au_2Cl_2, respectively.

Synthesis of the substrates

(*E*)-1-phenyl-4-oxa-1-hepten-6-yne[4]

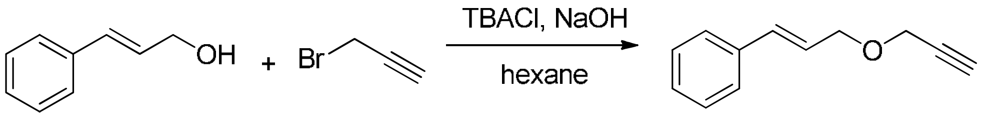

Cinnamyl alcohol (1.00 g, 7.45 mmol) was suspended in hexane (15 mL) and 50% aq. NaOH (2.98 g, 74.5 mmol) was added. The obtained suspension was vigorously stirred at RT for 10 min, and then Bu_4NCl (0.124 g, 0.48 mmol) was added followed by the dropwise addition of propargyl bromide (80 % wt. in toluene; 8.94 mmol, 1.06 g). The reaction mixture was vigorously stirred for 3 h at room temperature until total consumption of the alcohol (TLC monitoring). The reaction mixture was diluted with 30 mL of water, organic phase was separated and the aqueous phase was extracted with hexane (2×15 mL). The combined organic layers were washed with brine (15 mL), dried over $MgSO_4$ and concentrated. The residue was purified by flash chromatography (hexane/EtOAc = 5:1) to afford (*E*)-1-phenyl-4-oxa-1-hepten-6-yne in 97% yield as a colourless liquid. ^{1}H NMR (400 MHz, $CDCl_3$): δ 7.45–7.40 (m, 3H), 7.38–7.32 (m, 3H), 7.31–7.26 (m, 2H), 6.68 (dt, J = 16.1, 1.5 Hz, 1H), 6.31 (dt, J = 16.1, 6.2 Hz, 1H), 4.28 (dd, J = 6.2, 1.5 Hz, 2H), 4.24 (d, J = 2.4 Hz, 2H), 2.49 (t, J = 2.4 Hz, 1H).

Enynes **1a–c** were prepared from (*E*)-1-phenyl-4-oxa-1-hepten-6-yne in accordance with the published procedure.[5]

(*E*)-1-(3-cinnamyloxy)prop-1-ynyl)-4-nitrobenzene 1a[5]

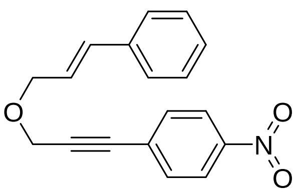

Pale-yellow solid; yield 84%; ^{1}H NMR (400 MHz, $CDCl_3$): δ 8.22–8.16 (m, 2H, $PhNO_2$), 7.63–7.55 (m, 2H, $PhNO_2$), 7.44–7.37 (m, 2H, Ph), 7.36–7.29 (m, 2H, Ph), 7.29–7.25 (m, 1H, Ph), 6.68 (d, *J* = 16.0 Hz, 1H), 6.32 (dt, *J* = 16.0, 6.3 Hz, 1H), 4.46 (s, 2H), 4.31 (dd, *J* = 6.3, 1.4 Hz, 2H). ^{13}C NMR (100 MHz, $CDCl_3$): δ 147.2 (Cq), 136.4 (Cq), 133.6, 132.5, 129.5 (Cq), 128.6, 128.0, 126.5, 124.9, 123.6, 90.7 (C≡C), 84.5 (C≡C), 70.8 (CH_2), 57.7 (CH_2).

(*E*)-1-(3-cinnamyloxy)prop-1-ynyl)-3-methoxybenzene 1b

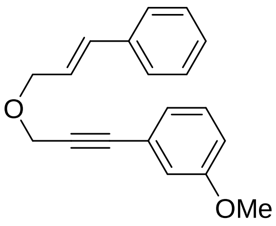

Colourless oil; yield 73%; R_f = 0.35 (hexane/EtOAc = 10:1); ^{1}H NMR (400 MHz, $CDCl_3$): δ 7.43–7.38 (m, 2H, Ph), 7.35-7.30 (m, 2H, Ph), 7.25–7.22 (m, 2H, Ph), 7.08–7.04 (m, 1H), 7.01–6.97 (m, 1H), 6.89 (dd, *J* = 8.1, 2.5 Hz, 1H), 6.68 (d, *J* = 15.9, 1H), 6.33 (dt, *J* = 15.9, 6.3 Hz, 1H), 4.33 (s, 2H), 4.31 (dd, J = 6.3, 1.2 Hz, 2H), 3.80 (s, 3H); ^{13}C NMR (100 MHz, $CDCl_3$): δ 159.2 (Cq), 136.5 (Cq), 133.3, 129.3, 128.5, 127.8, 126.5, 125.7, 125.2, 124.3, 123.6 (Cq), 116.6, 115.1, 86.3 (C≡C), 84.9 (C≡C), 70.4 (CH_2), 57.8 (CH_2), 55.2 (Me); IR (film) cm^{-1}: 3027 w, 2838 w, 2225 w, 1597 m, 1575 m, 1481 m, 1420m, 1449 m, 1354 m, 1319 m, 1289 s, 1202 m, 1164 m, 1072 m, 1041 s, 966 s, 854 m, 780 m, 744 s, 686 s, 616 m; HRMS (ESI) *m/z:* found: 279.1388, calculated for $C_{19}H_{19}O_2$ $[M+H]^+$: 279.1385.

(*E*)-4-[3-(3-phenyl-allyloxy)-prop-1-ynyl]-benzene 1c[5]

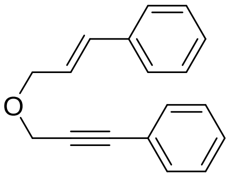

Colourless oil; yield 61%; ^{1}H NMR (400 MHz, $CDCl_3$): δ 7.50–7.36 (m, 4H), 7.36–7.29 (m, 4H), 7.28–7.23 (m, 2H), 6.68 (d, *J* = 15.9 Hz, 1H), 6.33 (dt, *J* = 15.9, 6.2 Hz, 1H), 4.44 (s, 2H), 4.29 (dd, J = 6.2, 1.4 Hz, 2H); ^{13}C NMR (100 MHz, $CDCl_3$): δ 136. 5 (Cq), 133.3, 131.8, 128.6, 128.4, 128.3, 127.8, 126.5, 125.7, 125.2, 122.6 (Cq), 109.9, 86.4 (C≡C), 85.0 (C≡C), 70.3 (CH_2), 57.9 (CH_2).

(*E*)-1-(3-cinnamyloxy)prop-1-ynyl)-4-methylbenzene 1d

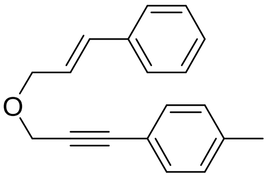

4-Methyliodobenzene (0.33 g, 1.51 mmol), $Pd(PPh_3)_2Cl_2$ (40 mg, 0.058 mmol), CuI (22 mg, 0.116 mmol) and NEt_3 (0.176 g, 1.74 mmol) were dissolved in dry THF (2 mL) under nitrogen. Then (*E*)-1-phenyl-4-oxa-1-hepten-6-yne was added dropwise and the reaction mixture was stirred at RT for 30 min (TLC monitoring). On completion, the reaction mixture was diluted with sat. aq. NH_4Cl (5 mL) and extracted with EtOAc (3×15 mL). The combined organic phases were washed with brine (10 mL), dried over $MgSO_4$, concentrated and purified on SiO_2 (hexane/EtOAc = 20:1) to afford **1d** as colourless oil. Yield 41%; R_f = 0.44 (hexane/EtOAc = 10:1); ^{1}H NMR (400 MHz, $CDCl_3$): δ 7.43–7.28 (m, 6H), 7.28–7.22 (m, 1H), 7.12 (d, *J* = 7.9 Hz, 2H), 6.67 (d, *J* = 15.9 Hz, 1H), 6.33 (dt, *J* = 15.9, 6.2 Hz, 1H), 4.42 (s, 2H), 4.30 (dd, *J* = 6.2, 1.2 Hz, 2H), 2.35 (s, 3H); ^{13}C NMR (100 MHz, $CDCl_3$): δ 138.6 (Cq), 136.6 (Cq), 133.3, 131.7, 129.0, 128.5, 127.8, 126.5, 125.3, 119.5 (Cq), 109.94, 86.5 (C≡C), 84.3 (C≡C), 70.3 (CH_2), 57.9 (CH_2), 21.5 (Me); IR

(film) cm^{-1}: 3027 w, 2847 w, 2236 w, 1719 w, 1509 m, 1449 w, 1353 m, 1258 w, 1105 m, 1072 s, 1021 m, 964 s, 815 s, 743 s, 691 s; HRMS (ESI) *m/z:* found: 263.1426, calculated for $C_{19}H_{19}O$ $[M+H]^+$: 263.1436.

Platinum-catalysed racemic cycloisomerisation

General procedure: A Radley's reaction tube was charged with a magnetic stir bar, $PtCl_2$ (5 mol%) and the corresponding enyne (1 mol equiv.). A PTFE screw cap was fitted and the reaction vessel was purged with N_2 before the addition of dry toluene (0.5 M) via syringe through the rubber septum. The tube was then positioned in a reaction carousel and left to stir at 80 °C for 24 h under N_2. Upon cooling to room temperature, the solvent was evaporated and the residue was purified by column chromatography (hexane/EtOAc = 9:1 or 8:2).

Gold(I)-catalysed enantioselective cycloisomerisation

General procedure: A Radley's reaction tube was charged with a magnetic stir bar and the corresponding enyne (1 mol equiv.). A PTFE screw cap was fitted and the reaction vessel was purged with N_2 before the addition of dry toluene via syringe through the rubber septum. In a separate vessel, a mixture of the requisite gold catalyst (5 mol%) and $AgBF_4$ (10 mol% or 5 mol%) in dry toluene was stirred at room temperature for 30 minutes, before it was added to the first reaction vessel via syringe through the rubber septum. The tube was then positioned in a reaction carousel and left to stir. Conversions were monitored by TLC and/or NMR integration. Upon completion, the solvent was evaporated and the residue was purified by column chromatography (hexanes/EtOAc = 9:1 or 8:2).

6-(4-nitrophenyl)-7-phenyl-3-oxabicyclo[4.1.0]hept-4-ene 2a

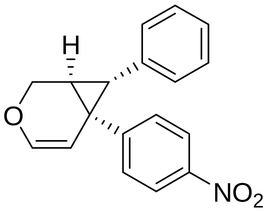

$[\alpha]_D^{20}$ = –3.0 (c 1.32, $CHCl_3$) at 64% *ee*; ^{1}H NMR (400 MHz, $CDCl_3$): δ 8.02 (d, J = 8.8 Hz, 2H), 7.25–7.19 (m, 2H), 7.11–7.02 (m, 3H), 6.85–6.67 (m, 2H), 6.32 (d, J = 6.0 Hz, 1H), 5.31 (dd, J = 5.9, 0.7 Hz, 1H), 4.44 (dd, J = 10.7, 1.0 Hz, 1H), 4.10 (dd, J = 10.6, 2.0 Hz, 1H), 2.90 (d, J = 6.1 Hz, 1H), 2.56 (d, J = 5.6 Hz, 1H). ^{13}C and mass spectroscopy data for compound **2c** were identical to the literature values.[5] HPLC conditions: Chiralpak OD-H column, 5% IPA in *n*-hexane, 1 mL/min, t_R = 10.1 and 14.7 min.

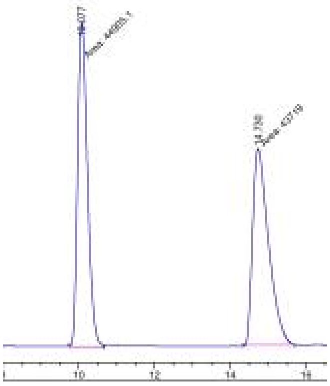

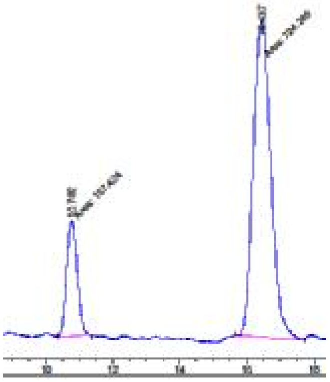

Peak #	RetTime [min]	Area %
1	10.789	17.9714
2	16.970	82.0286

64% *ee*

6-(3-methoxyphenyl)-7-phenyl-3-oxabicyclo[4.1.0]hept-4-ene 2b

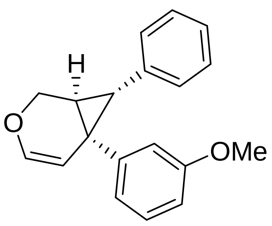

R_f = 0.41 (hexane/EtOAc = 10/1); $[\alpha]_D^{20}$ = –14.3 (c 0.42, $CHCl_3$) at 66% *ee*; ^{1}H NMR (400 MHz, $CDCl_3$): δ 7.11–7.01 (m, 4H), 6.83–6.78 (m, 2H), 6.72–6.63 (m, 2H), 6.60–6.56 (m, 1H), 6.26 (d, *J* = 6.0 Hz, 1H), 5.34 (dd, *J* = 5.9, 0.7 Hz, 1H), 4.40 (dd, *J* = 10.5, 1.1 Hz, 1H), 4.07 (dd, *J* = 10.5, 2.0 Hz, 1H), 3.64 (s, 3H), 2.76 (d, *J* = 6.0 Hz, 1H), 2.43 (d, *J* = 5.4 Hz, 1H); ^{13}C NMR (100 MHz, $CDCl_3$): δ 159.4 (Cq), 141.4 (Cq), 140.6 (CH), 137.6 (Cq), 129.1 (CH), 127.7 (2C), 127.6 (2C), 125.7 (CH), 122.1 (CH), 115.3 (CH), 112.1 (CH), 111.1 (CH), 61.3 (CH_2), 55.1 (CH_3), 37.3 (CH), 30.9 (Cq), 29.9 (CH); HRMS (CI-NH_3) *m/z*: found: 279.1385; calculated for $C_{19}H_{19}O_2$ $[M+H]^+$: 279.1393; HPLC conditions: Chiralpak OD-H column, 5% IPA in *n*-hexane, 1 mL/min, t_R = 5.4 and 6.3 min.

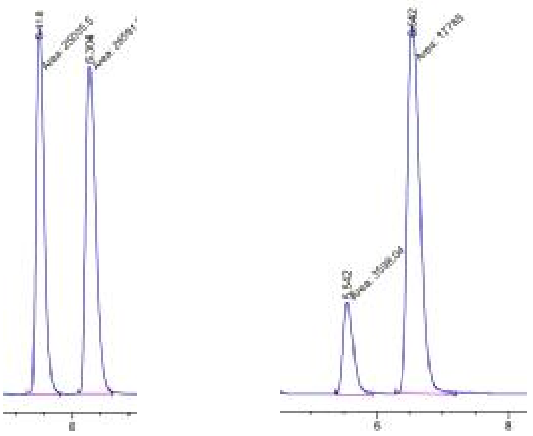

Peak #	RetTime [min]	Area %
1	5.542	16.8266
2	6.542	83.1734

66% *ee*

6,7-diphenyl-3-oxabicyclo[4.1.0]hept-4-ene 2c

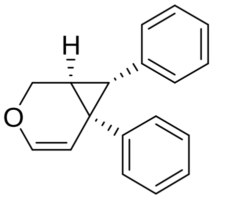

$[\alpha]_D^{20}$ = +3.3 (c 1.20, $CHCl_3$) at 78% *ee*;^{1}H NMR (400 MHz, $CDCl_3$): δ 7.31–7.00 (m, 10H), 6.27 (d, *J* = 6.0 Hz, 1H), 5.36 (d, *J* = 5.7 Hz, 1H), 4.42 (dd, *J* = 10.5, 1.0 Hz, 1H), 4.09 (dd, *J* = 10.5, 2.0 Hz, 1H), 2.78 (d, *J* = 5.9 Hz, 1H), 2.46 (d, *J* = 5.3 Hz, 1H); ^{13}C and mass spectroscopy data for compound **2c** were identical to the literature values;[6] HPLC conditions: Chiralpak OD-H column, 5% IPA in *n*-hexane, 1 mL/min, t_R = 4.7 and 5.1 min.

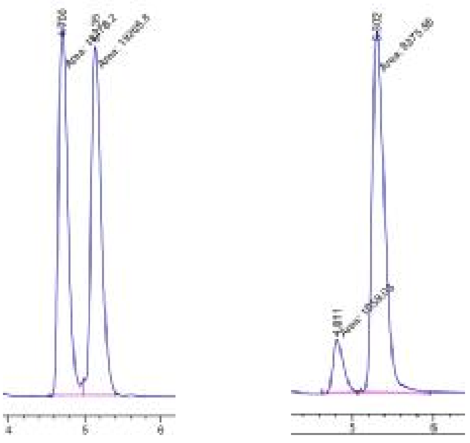

Peak #	RetTime [min]	Area %
1	4.811	11.2156
2	5.302	88.7844

78% *ee*

7-phenyl-6-(p-tolyl)-3-oxabicyclo[4.1.0]hept-4-ene 2d

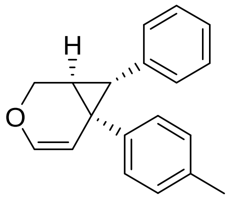

R_f = 0.53 (hexane/EtOAc = 10/1); $[\alpha]_D^{20}$ = +4.3 (c 0.96, $CHCl_3$) at 74% *ee*; ^1H NMR (400 MHz, $CDCl_3$): δ 7.06 (d, J = 7.4 Hz, 3H), 6.97 (s, 4H), 6.78 (dd, J = 7.9, 1.4 Hz, 2H), 6.25 (d, J = 6.0 Hz, 1H), 5.33 (dd, J = 5.9, 0.6 Hz, 1H), 4.40 (dd, J = 10.5, 1.0 Hz, 1H), 4.08 (dd, J = 10.5, 2.0 Hz, 1H), 2.74 (d, J = 6.0 Hz, 1H), 2.41 (d, J = 6.3 Hz, 1H), 2.25 (s, 3H); ^{13}C NMR (100 MHz, $CDCl_3$): δ 140.4 (OCH=, 137.8 (Cq), 136.7 (Cq), 135.9 (Cq), 129.5, 128.9, 127.6, 125.5, 111.8 (CH=), 77.3, 76.7, 61.4 (CH_2), 37.2, 30.6 (Cq), 29.9, 21.1; HRMS (CI-NH_3) *m/z*: found: 263.1436; calculated for $C_{19}H_{19}O$ $[M+H]^+$: 263.1435; HPLC conditions: Chiralpak OD-H column, 5% IPA in *n*-hexane, 1 mL/min, t_R = 4.5 and 5.4 min.

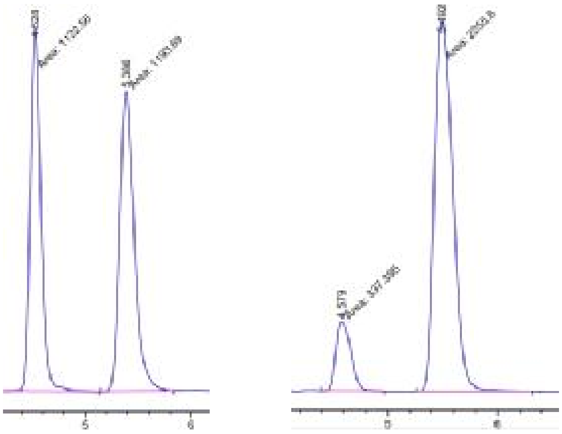

Peak #	RetTime [min]	Area %
1	4.579	13.0118
2	5.492	86.9882

74% *ee*

Crystal Structures:

Crystal data for [(**L10**)Au_2Cl_2]: $C_{38}H_{34}Au_2Cl_2N_2O_4P_2$, M = 1109.45, monoclinic, $P2_1$ (no. 4), a = 11.02454(10), b = 22.7167(2), c = 15.34786(15) Å, β = 102.9715(10)°, V = 3745.65(6) $Å^3$, Z = 4 (2 independent molecules), D_c = 1.967 g cm^{-3}, μ(Mo-Kα) = 8.095 mm^{-1}, T = 173 K, colourless tabular shards, Oxford Diffraction Xcalibur 3 diffractometer; 22285 independent measured reflections (R_{int} = 0.0244), F^2 refinement,[7] R_1(obs) = 0.0288, wR_2(all) = 0.0611, 20429 independent observed absorption-corrected reflections [$|F_o| > 4\sigma(|F_o|)$, $2\theta_{max}$ = 66°], 909 parameters. The structure of [(**L10**)AuCl]$_2$ was found to contain two crystallographically independent complexes (**A** and **B**) in the asymmetric unit, shown in figures S1 and S2 respectively. The absolute structure of [(**L10**)AuCl]$_2$ was determined by a combination of R-factor tests [R_1^+ = 0.0288, R_1^- = 0.0595] and by use of the Flack parameter [x^+ = 0.000(3), x^- = 1.009(3)]. CCDC 1005640.

Crystal data for [(**L11**)Au_2Cl_2]: $C_{46}H_{50}Au_2Cl_2N_2O_4P_2\cdot0.5(CHCl_3)$, M = 1281.34, monoclinic, $C2$ (no. 5), a = 22.5669(6), b = 12.4319(3), c = 18.9773(4) Å, β = 109.462(3)°, V = 5019.8(2) $Å^3$, Z = 4, D_c = 1.695 g cm^{-3}, μ(Mo-Kα) = 6.130 mm^{-1}, T = 173 K, colourless blocky needles, Oxford Diffraction Xcalibur 3 diffractometer; 16164 independent measured reflections (R_{int} = 0.0286), F^2 refinement,[7] R_1(obs) = 0.0377, wR_2(all) = 0.0843, 13441 independent observed absorption-corrected reflections [$|F_o| > 4\sigma(|F_o|)$, $2\theta_{max}$ = 65°], 571 parameters. The absolute structure of [(**L11**)Au_2Cl_2]was determined by a combination of R-factor tests [R_1^+ = 0.0377, R_1^- = 0.0644 and by use of the Flack parameter [x^+ = 0.000(5)]. CCDC 1005641.

The included chloroform solvent molecule in the structure of [(**L11**)AuCl]$_2$ was found to be slightly disordered and of partial occupancy. Inspection of the thermal parameters suggested an occupancy of approximately 50%, so it was fixed at exactly 50% for convenience. The disorder was handled by optimising the geometry of the sole orientation and allowing the thermal ellipsoids to expand to accommodate the slightly mis-aligned orientations. The non-hydrogen atoms were refined anisotropically.

Table S1. Selected conformational parameters for the structures of [(**L10**)Au_2Cl_2] (molecules **A** and **B**) and [(**L11**)Au_2Cl_2].

	[(**L10**)Au_2Cl_2]-**A**	[(**L10**)Au_2Cl_2]-**B**	[(**L11**)Au_2Cl_2]
$py_{(N4)}$–$py_{(N9)}$ (°)[a]	87.8	81.5	66.4
$py_{(N4)}$–[P1-Au1] (°)[b]	47.0	87.7	24.0
$py_{(N9)}$–[P2-Au2] (°)[b]	47.1	28.5	25.3
Au1···Au2 (Å)	3.6701(3)	3.1821(2)	4.7921(4)

[a] The torsion angle about the central π–π bond. [b] The torsion angle about the designated π–P bond as measured by the mean of the normalised C–C–P–Au angles.

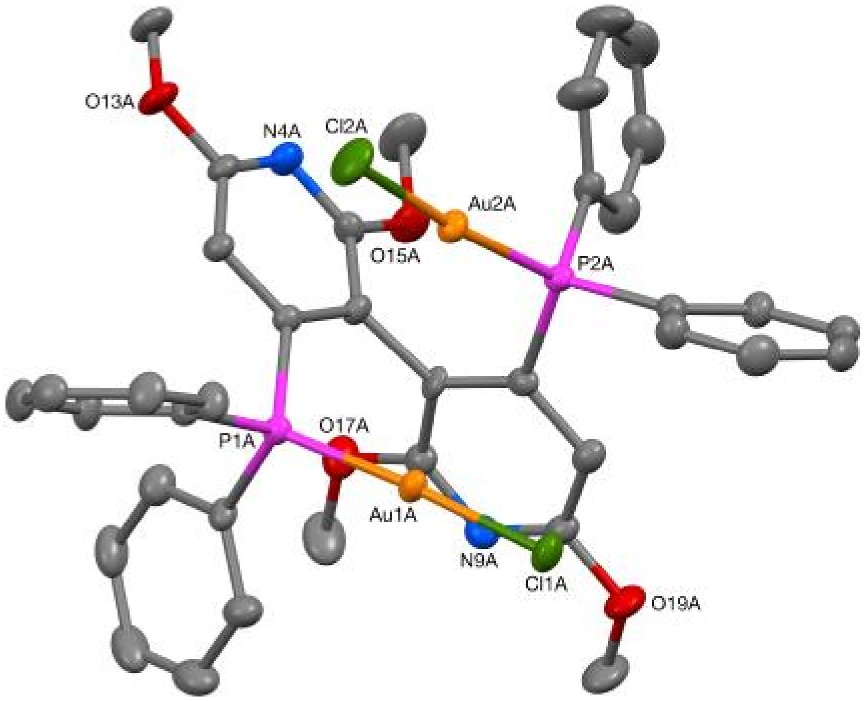

Fig. S1 The structure of one (**A**) of the two independent complexes present in the crystals of [(**L10**)Au_2Cl_2] (50% probability ellipsoids). Selected bond lengths (Å) and angles (°); Au(1A)–Cl(1A) 2.2817(12), Au(1A)–P(1A) 2.2290(12), Au(2A)–Cl(2A) 2.2773(12), Au(2A)–P(2A) 2.2310(12), Cl(1A)–Au(1A)–P(1A) 176.34(5), Cl(2A)–Au(2A)–P(2A) 175.00(5). The Au(1A)···Au(2A) separation is 3.6701(3) Å.

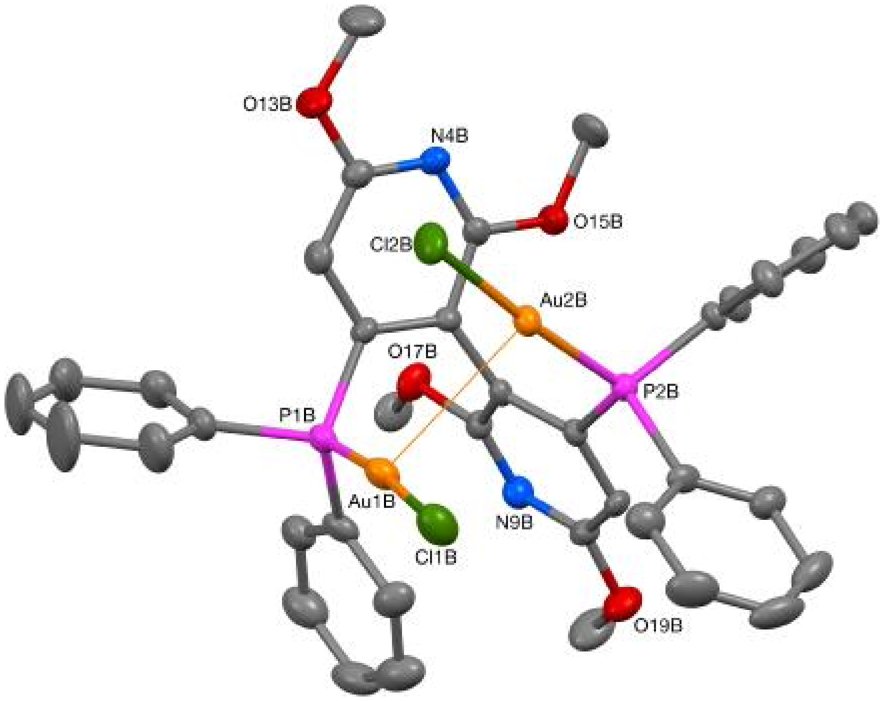

Fig. S2 The structure of one (**B**) of the two independent complexes present in the crystals of [(**L10**)Au_2Cl_2] (50% probability ellipsoids). Selected bond lengths (Å) and angles (°); Au(1B)–Cl(1B) 2.2951(15), Au(1B)–P(1B) 2.2421(14), Au(2B)–Cl(2B) 2.2902(14), Au(2B)–P(2B) 2.2343(12), Cl(1B)–Au(1B)–P(1B) 173.75(5), Cl(2B)–Au(2B)–P(2B) 176.18(5). The Au(1B)···Au(2B) separation is 3.1821(2) Å.

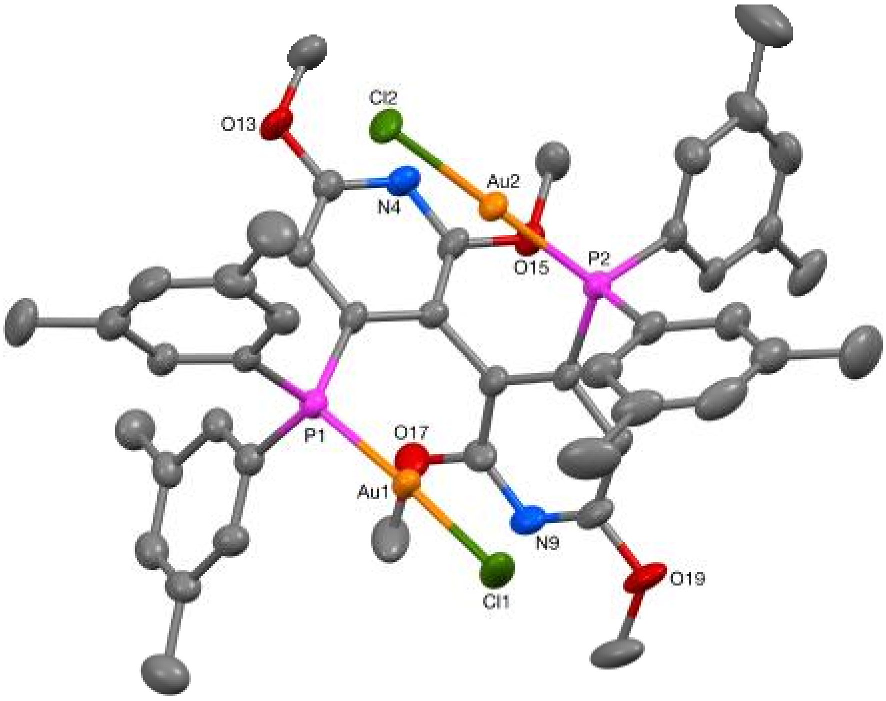

Fig. S3 The crystal structure of [(**L11**)Au_2Cl_2] (50% probability ellipsoids). Selected bond lengths (Å) and angles (°); Au(1)–Cl(1) 2.2740(15), Au(1)–P(1) 2.2304(15), Au(2)–Cl(2) 2.2815(13), Au(2)–P(2) 2.2317(12), Cl(1)–Au(1)–P(1) 176.96(6), Cl(2)–Au(2)–P(2) 179.31(6). The Au(1)···Au(2) separation is 4.7921(4) Å.

References

1. Y. Zhu, C. S. Day, L. Zhang, K. J. Hauser, A. C. Jones, *Chem. Eur. J.*, **2013**, *19*, 12264–12271.
2. A. D. Melhado, M. Luparia, F. D. Toste, *J. Am. Chem. Soc.*, **2007**, *129*, 12638–12639.
3. J. W. Bats, M. Hamzic, A. S. K. Hashmi, *Acta Crystallogr. Sect. E*, **2007**, *63*, m2344.
4. Trost, B. M., Edstrom, E. D., Carter-Petillo, M.B. *J. Org. Chem.*, **1989**, *54*, 4489–4490.
5. Kim, H., Lee, C. *Org. Lett.*, **2002**, *4*, 4369–4372.
6. Blum, J., Beer-Kraft, H., Badrieh, Y. *J. Org. Chem.*, **1995**, *60*, 5567–5569.
7. SHELTL, Bruker AXS, Madison, WI; SKELX-97, G.M. Sheldrick, Acta Cryst., **2008**, *A64*, 112-122.

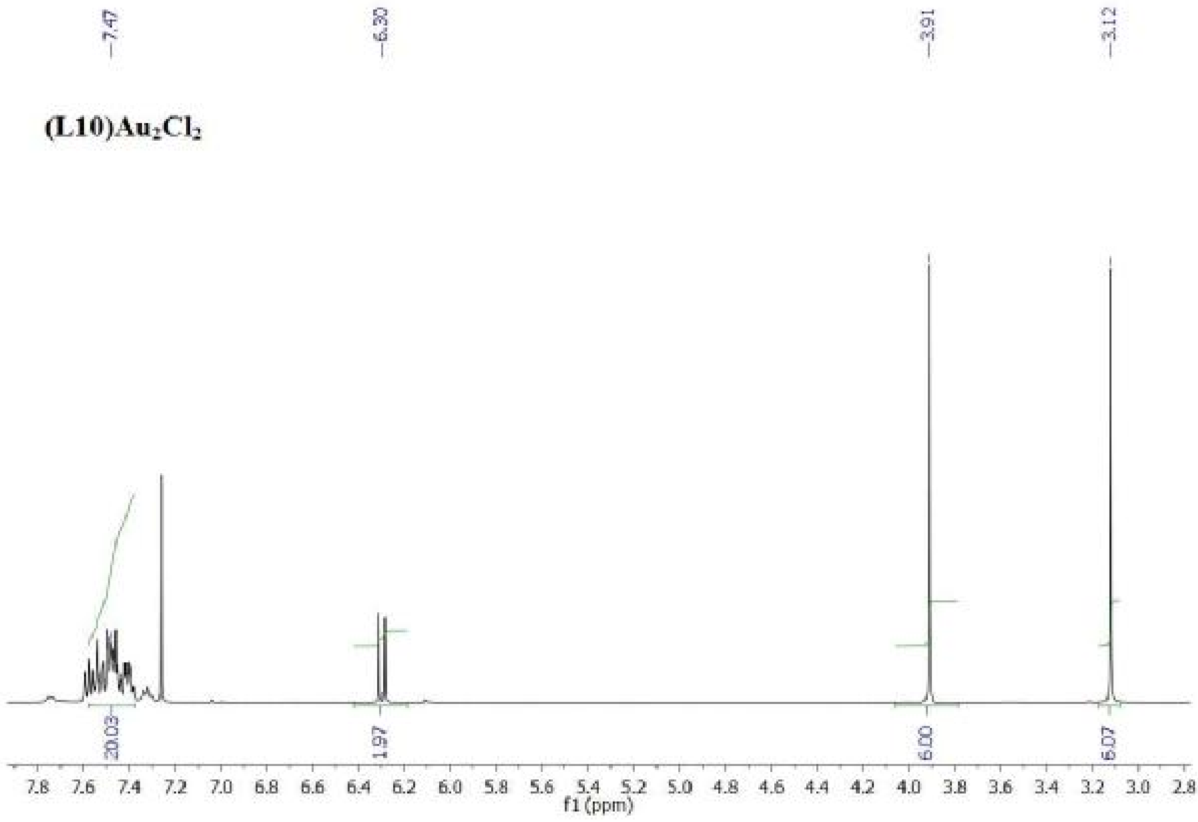
7.47
6.30
3.91
3.12
(L10)Au2Cl2
20.03
1.97
6.00
6.07
7.8 7.6 7.4 7.2 7.0 6.8 6.6 6.4 6.2 6.0 5.8 5.6 5.4 5.2 5.0 4.8 4.6 4.4 4.2 4.0 3.8 3.6 3.4 3.2 3.0 2.8
f1 (ppm)

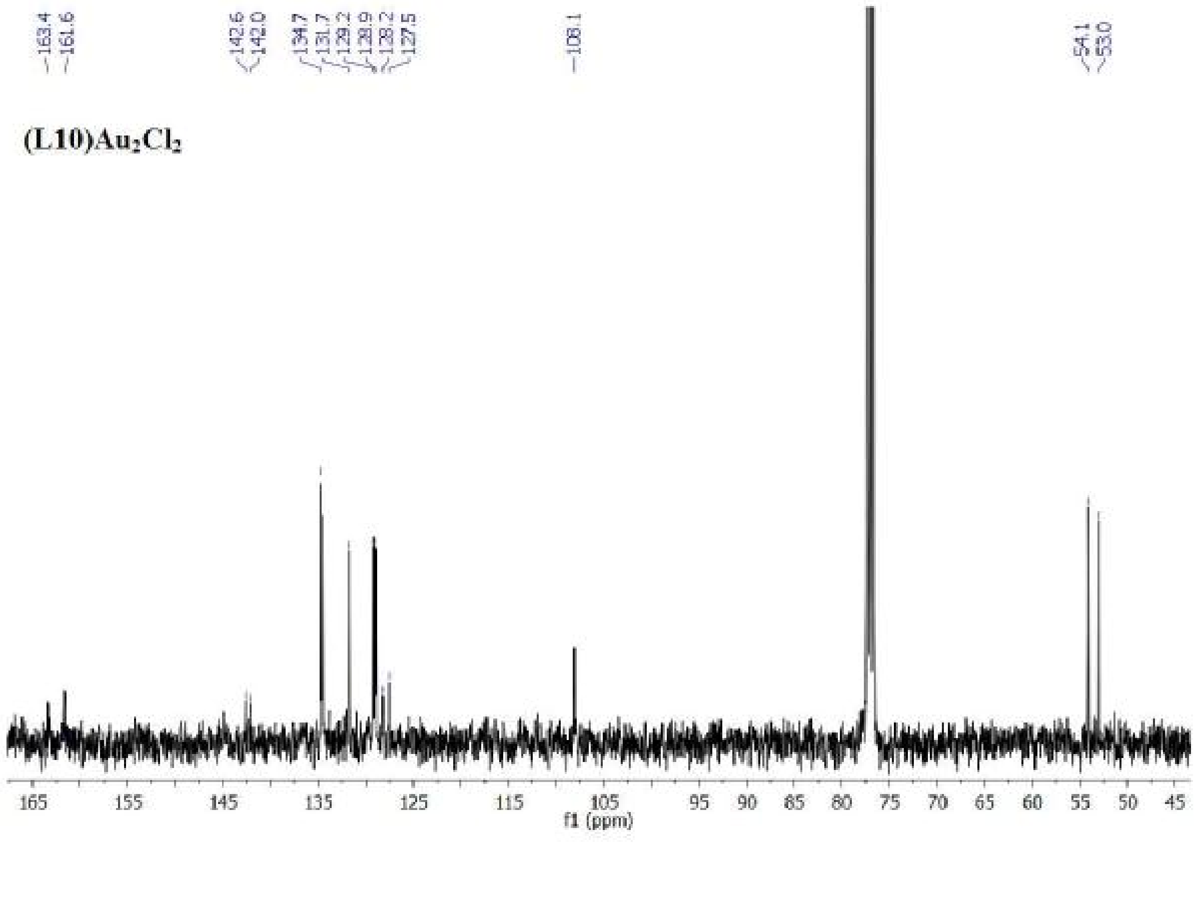
163.4
161.6
142.6
142.0
134.7
131.7
129.2
128.9
128.2
127.5
108.1
54.1
53.0
(L10)Au2Cl2
165 155 145 135 125 115 105 95 90 85 80 75 70 65 60 55 50 45
f1 (ppm)

(L10)Au_2Cl_2

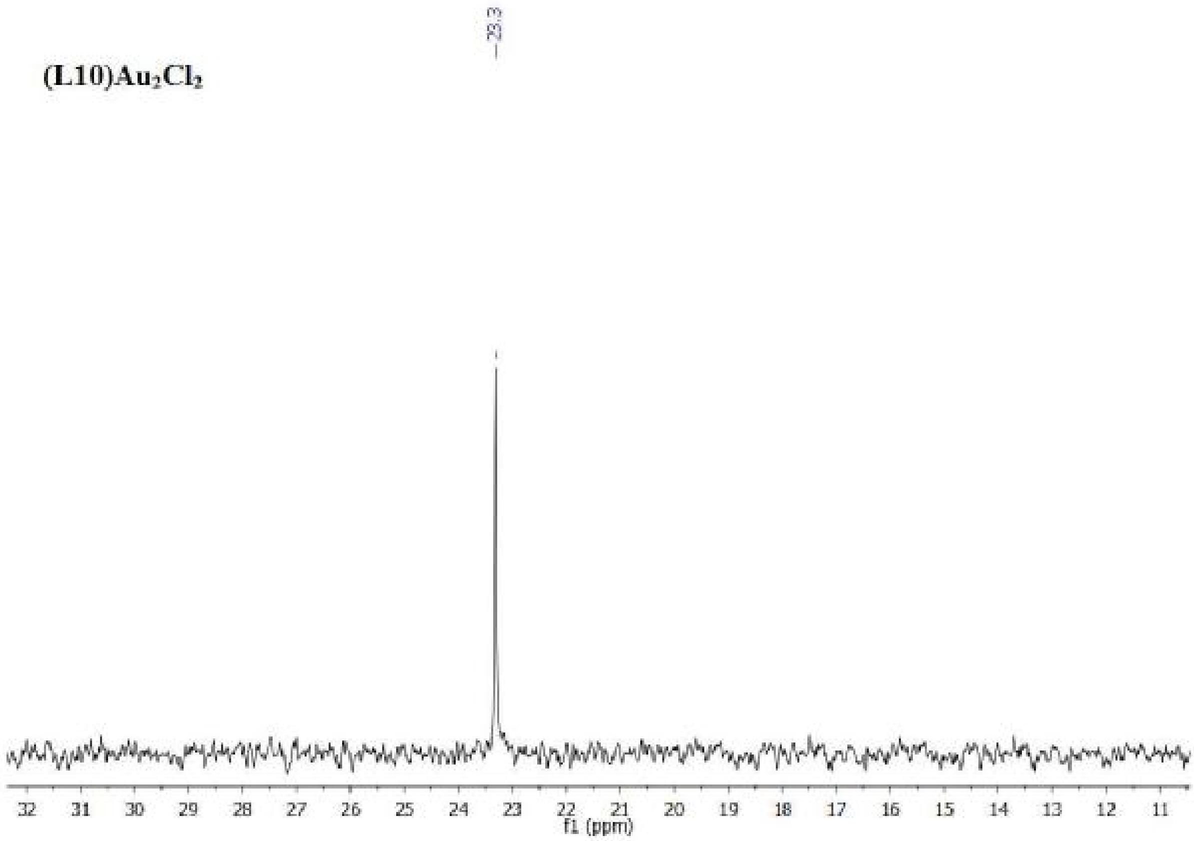

(L11)Au_2Cl_2

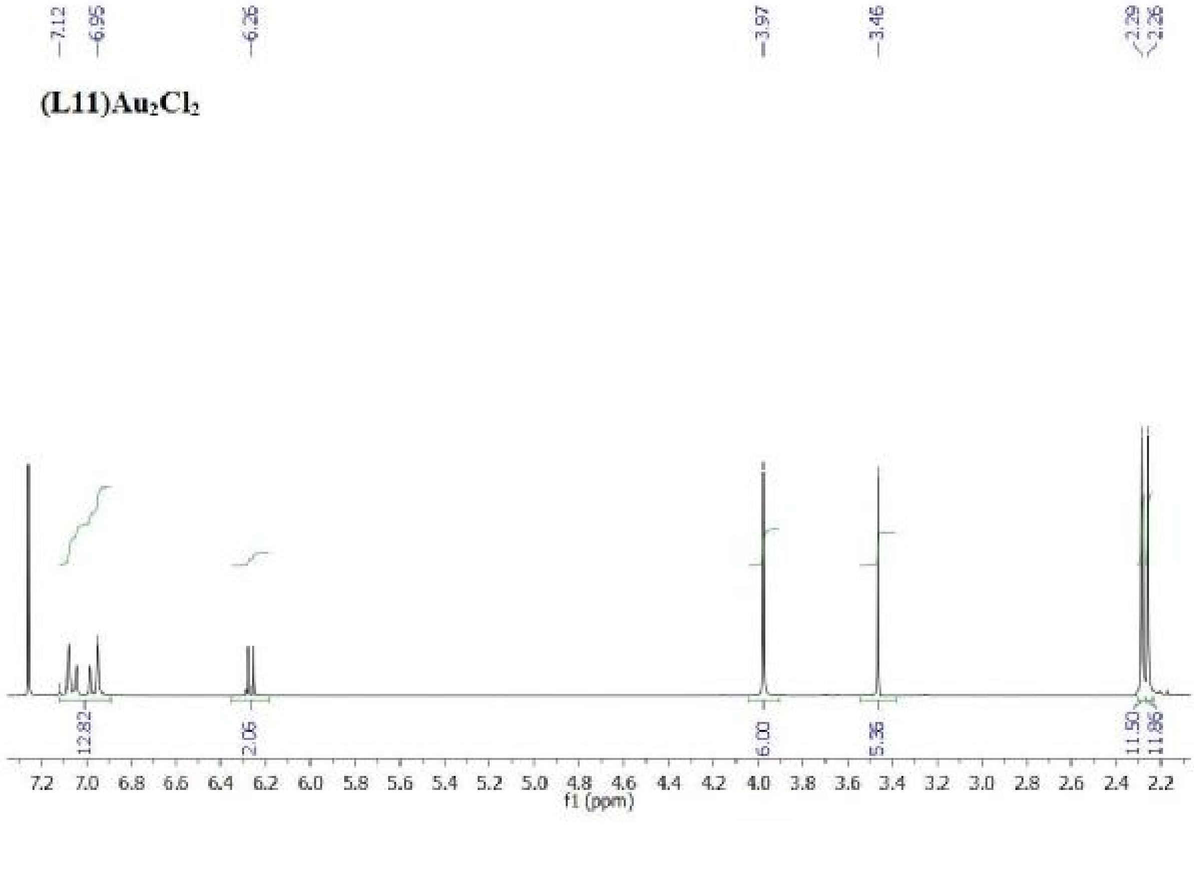

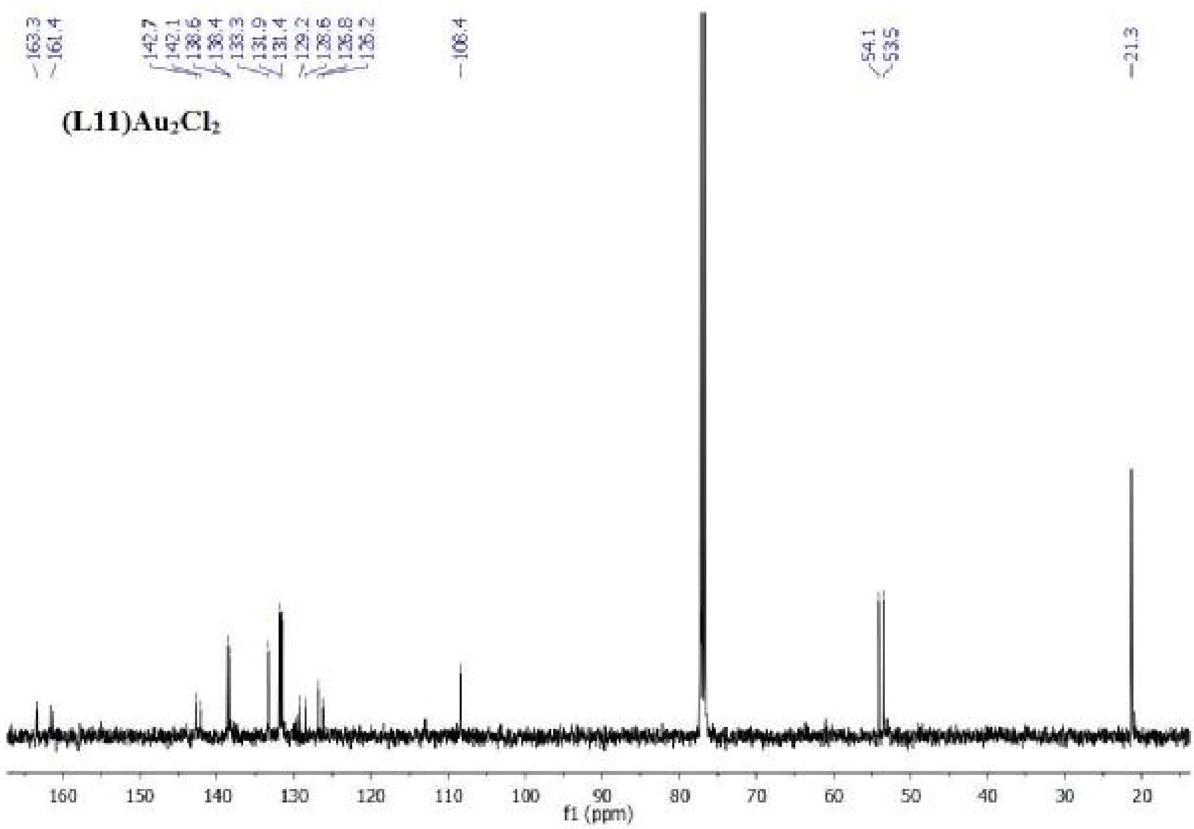
163.3
161.4
142.7
142.1
138.6
138.4
133.3
131.9
131.4
129.2
128.6
126.8
126.2
108.4
54.1
53.5
21.3
(L11)Au2Cl2
160
150
140
130
120
110
100
90
80
70
60
50
40
30
20
f1 (ppm)

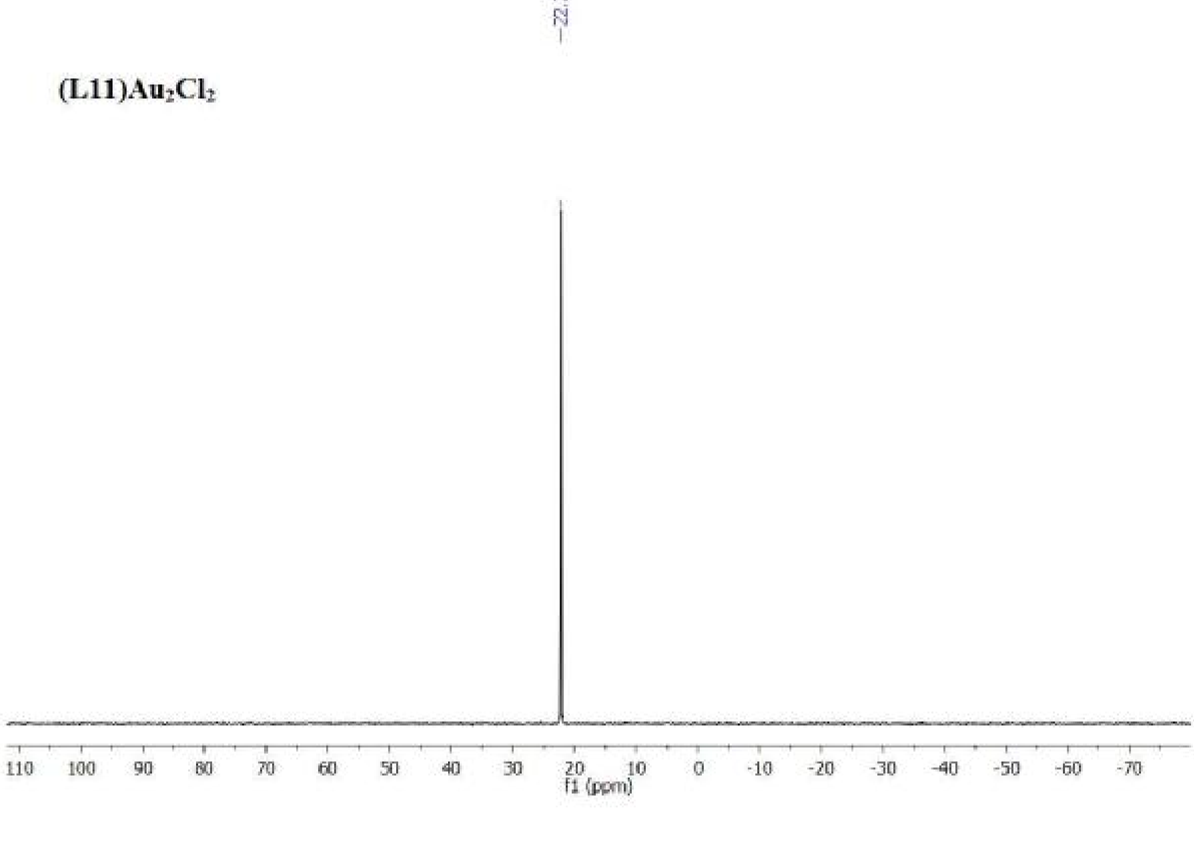
22.2
(L11)Au2Cl2
110
100
90
80
70
60
50
40
30
20
10
0
-10
-20
-30
-40
-50
-60
-70
f1 (ppm)

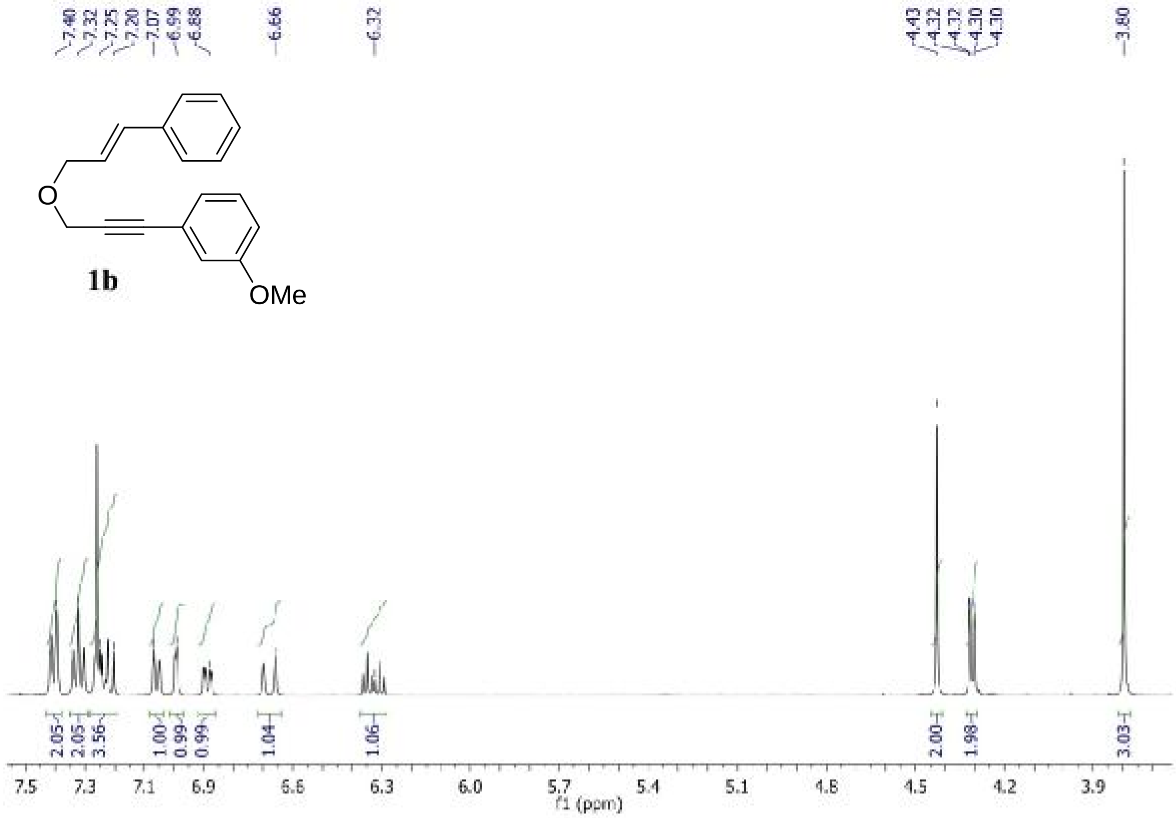
7.40
7.32
7.25
7.20
7.07
6.99
6.88
6.66
6.32
4.43
4.32
4.32
4.30
4.30
3.80
O
1b
OMe
2.05
2.05
3.56
1.00
0.99
0.99
1.04
1.06
2.00
1.98
3.03
7.5 7.3 7.1 6.9 6.6 6.3 6.0 5.7 5.4 5.1 4.8 4.5 4.2 3.9
f1 (ppm)

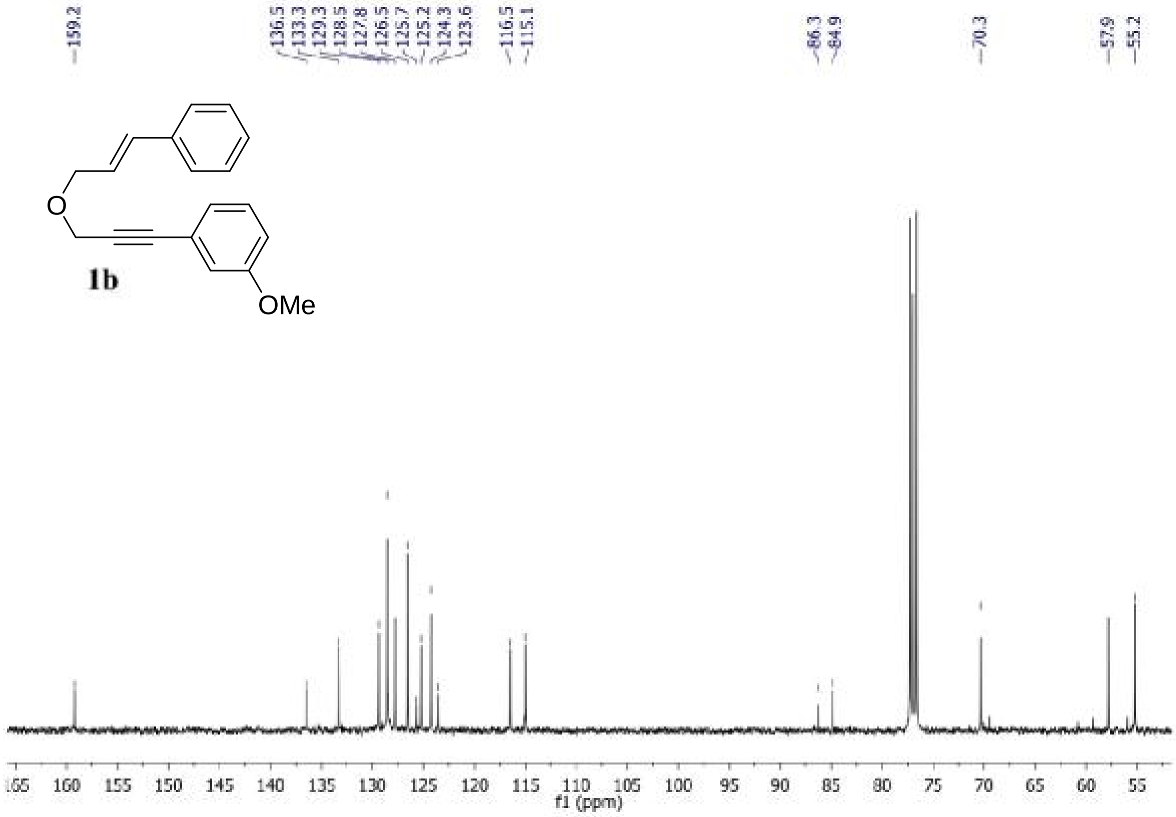
159.2
136.5
133.3
129.3
128.5
127.8
126.5
125.7
125.2
124.3
123.6
116.5
115.1
86.3
84.9
70.3
57.9
55.2
O
1b
OMe
165 160 155 150 145 140 135 130 125 120 115 110 105 100 95 90 85 80 75 70 65 60 55
f1 (ppm)

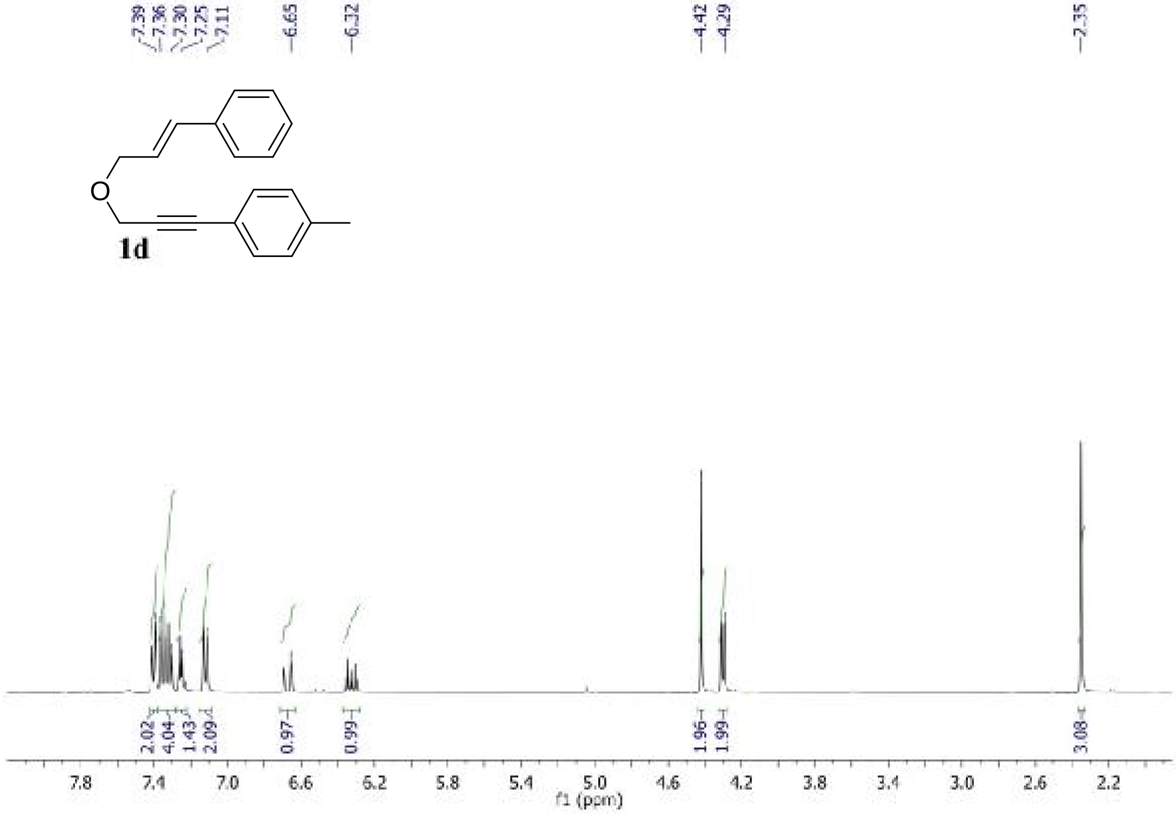
7.39
7.36
7.30
7.25
7.11
6.65
6.32
4.42
4.29
2.35
O
1d
2.02
4.04
1.43
2.09
0.97
0.99
1.96
1.99
3.08
f1 (ppm)

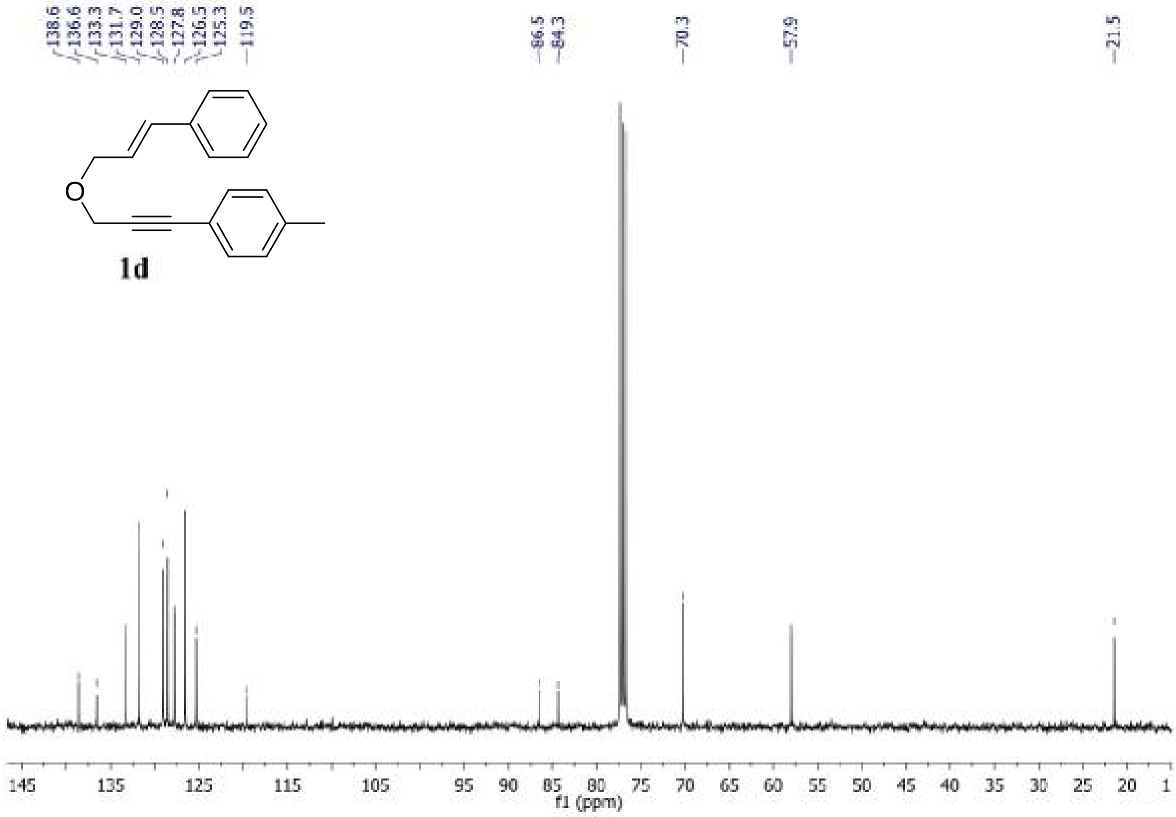
138.6
136.6
133.3
131.7
129.0
128.5
127.8
126.5
125.3
119.5
86.5
84.3
70.3
57.9
21.5
O
1d
f1 (ppm)

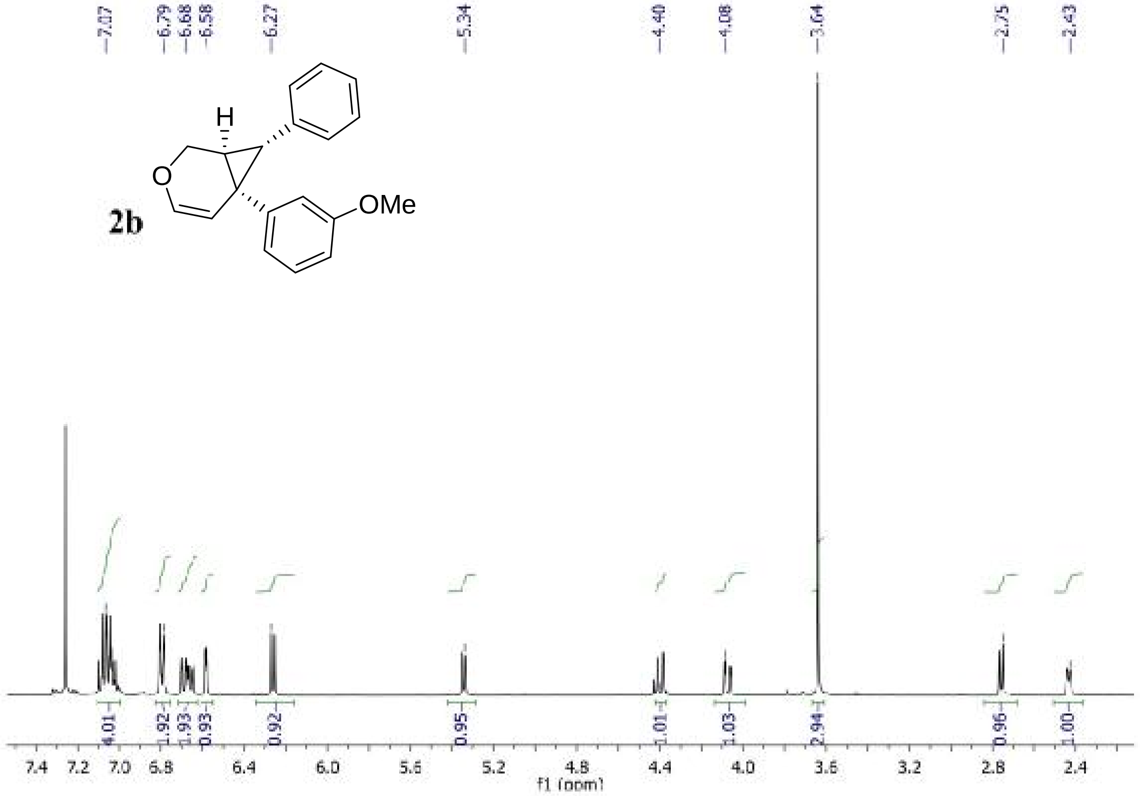
7.07
6.79
6.68
6.58
6.27
5.34
4.40
4.08
3.64
2.75
2.43
H
O
OMe
2b
4.01
1.92
1.93
0.93
0.92
0.95
1.01
1.03
2.94
0.96
1.00
7.4 7.2 7.0 6.8 6.4 6.0 5.6 5.2 4.8 4.4 4.0 3.6 3.2 2.8 2.4
f1 (ppm)

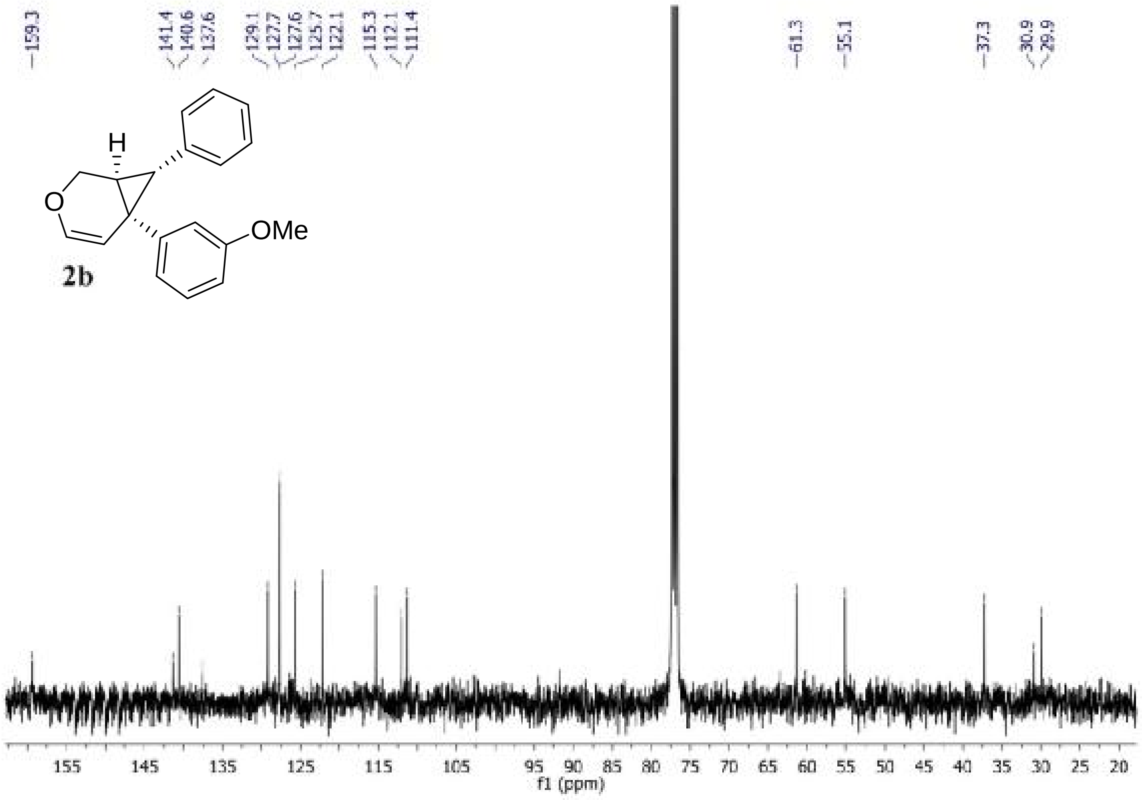
159.3
141.4
140.6
137.6
129.1
127.7
127.6
125.7
122.1
115.3
112.1
111.4
61.3
55.1
37.3
30.9
29.9
H
O
OMe
2b
155 145 135 125 115 105 95 90 85 80 75 70 65 60 55 50 45 40 35 30 25 20
f1 (ppm)

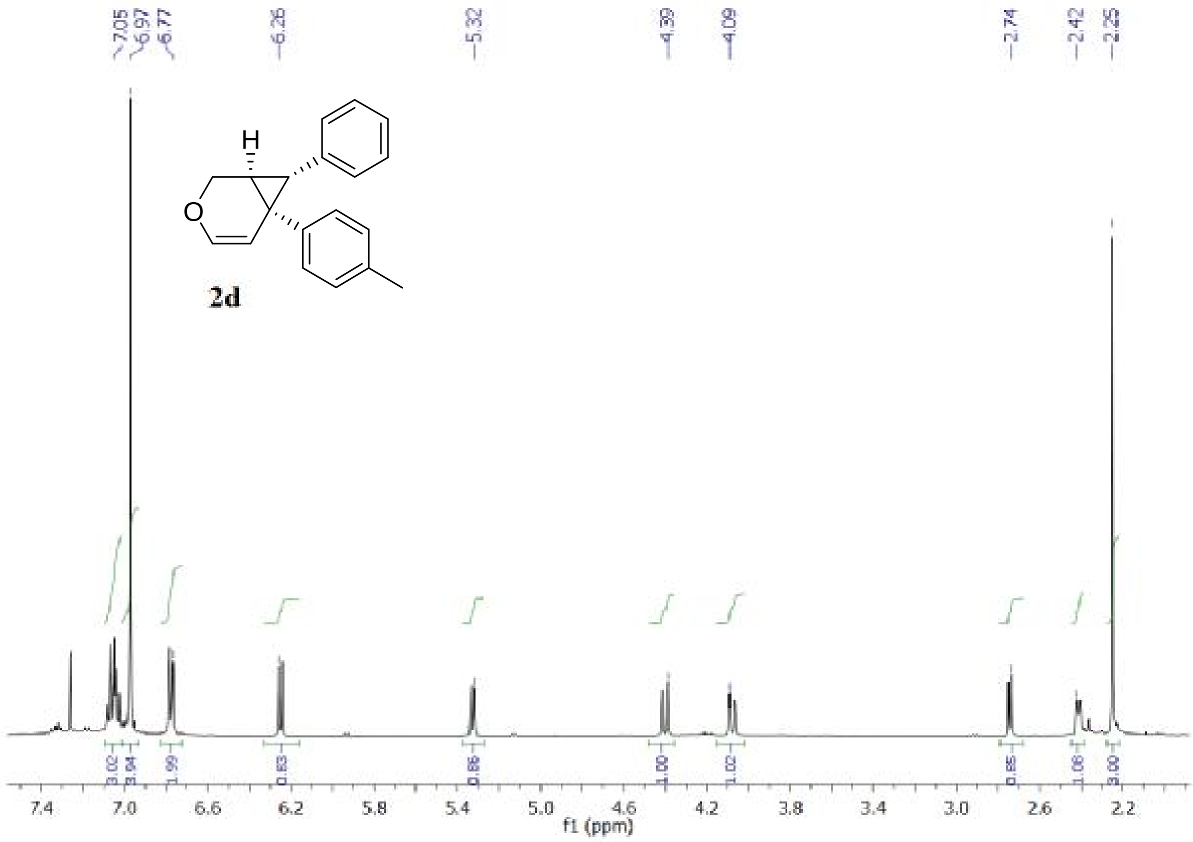
7.05
6.97
6.77
6.26
5.32
4.39
4.09
2.74
2.42
2.26
2d
3.02
3.04
1.99
0.83
0.86
1.00
1.02
0.85
1.06
3.00
7.4 7.0 6.6 6.2 5.8 5.4 5.0 4.6 4.2 3.8 3.4 3.0 2.6 2.2
f1 (ppm)

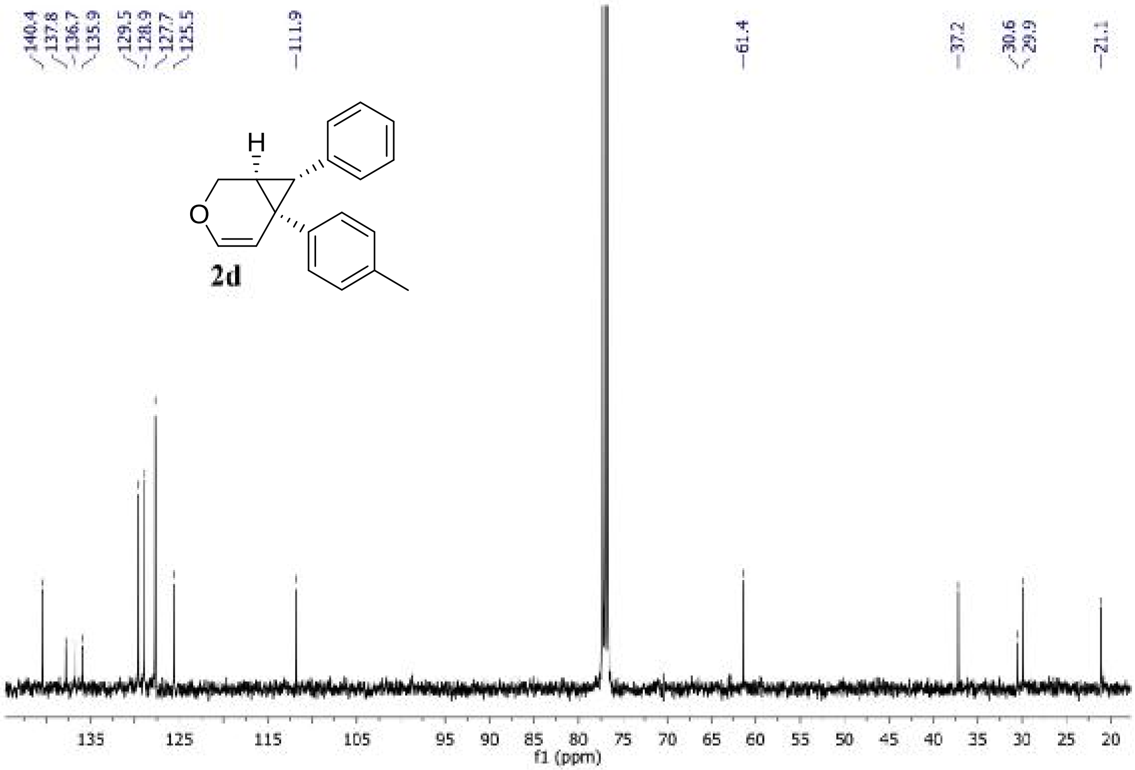
140.4
137.8
136.7
135.9
129.5
128.9
127.7
125.5
111.9
61.4
37.2
30.6
29.9
21.1
2d
135 125 115 105 95 90 85 80 75 70 65 60 55 50 45 40 35 30 25 20
f1 (ppm)
